# MYL9 expressed in cancer-associated fibroblasts regulate the immune microenvironment of colorectal cancer and promotes tumor progression in an autocrine manner

**DOI:** 10.1186/s13046-023-02863-2

**Published:** 2023-11-06

**Authors:** Shenghe Deng, Denglong Cheng, Jun Wang, Junnan Gu, Yifan Xue, Zhenxing Jiang, Le Qin, Fuwei Mao, Yinghao Cao, Kailin Cai

**Affiliations:** 1 Center for Liver Transplantation, Union Hospital, Tongji Medical College, Huazhong University of Science and Technology, Wuhan, 430022 China; 2 Department of Gastrointestinal Surgery, Union Hospital, Tongji Medical College, Huazhong University of Science and Technology, Wuhan, 430022 China; 3 Department of Digestive Surgical Oncology, Cancer Center, Union Hospital, Tongji Medical College, Huazhong University of Science and Technology, Wuhan, 430022 China

**Keywords:** MYL9, Cancer-associated fibroblasts, Colorectal cancer, Immunosuppressive microenvironment, Metastasis, Epithelial-mesenchymal transition

## Abstract

**Background:**

The tumor microenvironment (TME) is an important factor that regulates the progression of colorectal cancer (CRC). Cancer-associated fibroblasts (CAFs) are the main mesenchymal cells in the TME and play a vital role in tumor progression; however, the specific underlying mechanisms require further study.

**Methods:**

Multiple single-cell and transcriptome data were analyzed and validated. Primary CAFs isolation, CCK8 assay, co-culture assay, western blotting, multiple immunofluorescence, qRT-PCR, ELISA, immunoprecipitation, ChIP, double luciferase, and animal experiments were used to explore the potential mechanism of MYL9 regulation in CRC.

**Results:**

Our findings revealed that MYL9 was predominantly localized and expressed in CAFs rather than in CRC cells, and bioinformatics analysis revealed that high MYL9 expression was strongly associated with poor overall and disease-free survival in various tumors. In addition, high MYL9 expression is closely associated with M2 macrophage infiltration, which can lead to an immunosuppressive microenvironment in CRC, making it insensitive to immunotherapy. Mechanically, MYL9 can regulate the secretion of CAFs on CCL2 and TGF-β1, thus affecting the immune microenvironment and progression of CRC. In addition, MYL9 bounded with IQGAP1 to regulate CCL2 and TGF-β1 secretion through the ERK 1/2 pathway, and CCL2 and TGF-β1 synergistically promoted CRC cells progression through the PI3K-AKT pathway. Furthermore, MYL9 promotes epithelial-mesenchymal transition (EMT) in CRC. During the upstream regulation of MYL9 in CAFs, we found that the EMT transcription factor ZEB1 could bind to the MYL9 promoter in CAFs, enhancing the activity and function of MYL9. Therefore, MYL9 is predominantly expressed in CAFs and can indirectly influence tumor biology and EMT by affecting CAFs protein expression in CRC.

**Conclusions:**

MYL9 regulates the secretion of cytokines and chemokines in CAFs, which can affect the immune microenvironment of CRC and promote CRC progression. The relationship between MYL9 expression and CRC clinical staging and immunotherapy is closer in CAFs than in tumor cells; therefore, studies using CAFs as a model deserve more attention when exploring tumor molecular targets in clinical research.

**Supplementary Information:**

The online version contains supplementary material available at 10.1186/s13046-023-02863-2.

## Background

Colorectal cancer (CRC) is the third most common cancer worldwide, with an average 5-year overall survival (OS) rate of 60%, which is significantly lower in patients with advanced CRC [1]. Despite improvements in the survival rates of patients diagnosed with metastatic disease over the past 20 years, significant heterogeneity in survival outcomes remains [2]. Although many studies have found that interactions between different cells can promote the progression and metastasis of CRC, the underlying mechanism is complex and remains unclear.

Tumor microenvironment (TME) is closely associated with the occurrence and development of CRC. The TME is an aggregate composed of various cells and tissue structures, including tumor cells, immune-related cells, cancer-associated fibroblasts (CAFs), extracellular matrix (ECM), blood vessels, and lymphatic vessels [3]. The TME is involved in the activation and recruitment of immune cells, angiogenesis, and extracellular matrix reconstruction and is an important factor regulating the occurrence and development of cancer. This is closely related to patient prognosis and is a vital direction for research on therapeutic targets [4]. In CRC, stromal cells are activated in the TME and increase the secretion of collagen and collagen-remodeling enzymes, leading to continuous recombination of the ECM.

CAFs, including cancer-associated mesenchymal stem cells, are the most abundant stromal cells in the TME and play multiple roles in cancer cells and stroma through direct cell contact or paracrine cytokines, thus promoting ECM deposition and remodeling, extensive crosstalk with cancer cells, epithelial-mesenchymal transition (EMT), invasion, metastasis, and therapy resistance [5–7]. Ren et al. [8] found that exosome H19 secreted by CAFs acts as a competing endogenous RNA sponge of miR-141 that activate the β-catenin pathway and promote the development of CRC and chemotherapy resistance. Paauwe et al. [9] found that CAFs expressing endoglin can promote the progression and metastasis of CRC. Moreover, it could inhibit CAFs invasion and tumor metastasis after treatment with its specific antibody. In addition, some biomacromolecules can be expressed in both tumor cells and CAFs, and cellular communication between tumor cells and CAFs and their effects on the biological phenotype of tumors deserve attention.

MYL9, also known as MLC2 and MRLC1, is a protein-encoding gene that has been considered to play an important role in various cancers in recent years [10]. The high expression of MYL9 in breast cancer, esophageal squamous cell carcinoma, liver cancer, and epithelial ovarian cancer is associated with poor prognosis, while the low expression of MYL9 in non-small cell lung cancer, bladder cancer, and prostate cancer is associated with poor prognosis [11, 12]. However, we found that in CRC tissues, its expression was significantly dominant in CAFs; therefore, we further explored how MYL9 expression affects the biological function of tumors. Additionally, we explored novel therapeutic targets for CRC and identified potential biomarkers for immunotherapy and prognosis.

## Methods

### CRC tissue specimen and ethical statement

 Twenty pairs of fresh human CRC specimens and normal tissues were collected from the Wuhan union hospital (Supplementary file 1: Table 1). All patients did not receive adjuvant chemotherapy before surgery, and all patients signed informed consent. Studies involving human participants were reviewed and approved by the Ethics Committee and the Institutional Review Committee of Wuhan Union Medical College. All animal studies were carried out under the guidelines of Tongji Medical College of Huazhong University of Science and Technology and approved by the Animal Ethics Committee of Tongji Medical College of Huazhong University of Science and Technology.

### Isolation of primary CAFs and condition medium (CM) collection

Fresh colorectal adenocarcinoma and adjacent normal tissues were cleaned 4–6 times with sterile phosphate-buffered saline (PBS) containing 3% penicillin/streptomycin/amphozone and cut into 1 × 1 × 1 mm^3^ pieces using scissors. Digestion was then performed at 37 ℃ with 2% collagenase IV (Sigma, USA), 1% hyaluronidase (BioFroxx, Germany), and DNA I enzyme (BioFroxx, Germany) for 4–6 h. After digestion, the mixture was filtered through a 40-micron mesh (BD Falcon, USA) and centrifuged. Red blood cells were removed from the red blood cell lysis buffer and cultured in medium. After successful cell expansion, a-SMA and vimentin were used for immunofluorescence identification.

In CM, the cells were collected and inoculated into six-well plates for culturing. When the cells grew to 90%, fresh F12 medium without serum was used to replace the medium, and the culture was continued for 24 h. CM was collected and filtered, and stored at -80 ℃.

### Cell culture, reagents, and transfection

The colon cancer cell lines LoVo and SW480 and the normal intestinal epithelial cell line NCM460 were purchased from the American Type Culture Collection (ATCC, USA). These cell lines and primary CAF were cultured in Dulbecco’s modified Eagle’s medium (DMEM) (Invitrogen, USA) with a high glucose content, 10% fetal bovine serum (FBS) (Gibco, USA), and 1% penicillin/streptomycin. Cells were cultured at 37 ℃, under 5% CO_2_ and 95% air. The recombinant proteins including TGF-β1 (PeproTech, USA), CCL2 (PeproTech, USA), and LY294002 (SELLECK, USA) were purchased from their respective companies. MYL9 overexpressing plasmid and lentiviral shRNA particles were purchased from GeneChem Co. (Shanghai, China). Small interfering RNA were purchased from ReboBio Co. (Guangzhou, China).

Lipofectamine 2000 (Invitrogen, USA) and Opti-MEM (Invitrogen, USA) were used for transfection. Therefore, Lipofectamine 2000 was used to transfect the plasmids and small interfering RNA into primary CAFs. After 6 h of incubation, the Opti-MEM was replaced with fresh DMEM containing 10% FBS.

Lentiviral transfection was performed according to the manufacturer’s instructions; 5000 cells/well were seeded in 96-well cell culture plates, and lentiviral particles with a multiplicity of infection (MOI) of 10 were infected the next day in the presence of 10 mg/mL polybrene (Meilunbio, CHN). They were selected using 5 μg/mL purinamycin (Medchem express, USA). The shRNA sequences are listed in Supplementary file 1: Table 2.

### Western blotting, immunohistochemistry (IHC), immunofluorescence, multiplexed immunofluorescence, and co-immunoprecipitation

Tissues or cells were lysed on ice using radioimmunoprecipitation assay lysis buffer containing 1% protease and phosphatase inhibitors. The cell lysate products were centrifuged (4 ℃, 12000 rpm, 15 min) and the supernatant was collected. Protein content was determined using bicinchoninic acid assay. The obtained protein was added into 5 × loading buffer and heated at 95 ℃ for 10 min, and used for Western blot analysis. For co-immunoprecipitation (Co-IP) assay, the protein supernatant was collected in the same way as described above. After adding the target antibody and Protein A + G agarose (Beyotime, P2012) to the collected protein supernatant, the agarose beads were cleaned overnight at 4 ℃, then loading buffer was added and heated at 95 ℃ for 10 min.

For IHC assay, the paraffin specimens were sectioned, treated with H_2_O_2_ and non-specific antigen blocking, and the target antibody was added and stored at 4 ℃ overnight, followed by incubation with the secondary antibody. Signals were detected using a DAB staining kit (Solarbio, CHN, DA1016). For the immunohistochemical analysis, five visual fields were randomly selected from each section. The integrated optical density (IOD)/area value of each visual field was calculated using the ImageJ, and the average value was used for comparative analysis. For immunohistochemical quantitative analysis of immune cells, QuPath software was used to calculate the percentage of positive cells. The immunofluorescence method for tissue or cells is similar, but requires incubation under dark conditions and observation using fluorescence microscope or confocal microscope after 4,6-diamidino-2-phenylindole (DAPI) staining.

Multiplexed immunofluorescence was performed as follows: The CRC sections were dewaxed for antigen repair and underwent endogenous enzyme blocking with H_2_O_2_ for 30 min. It was then blocked with 10% goat serum (37 ℃, 30 min). The primary antibody was diluted according to the experimental concentration and incubated at 4 ℃ overnight. Horseradish peroxidase (HRP) enzyme-labeled secondary antibody was prepared using PBS with Tween 20 (PBST) (37 ℃, 1 h). Tyramide Signal Amplification (TSA) reagent was added to the tissue (37 ℃, 1 h) for antigen repair and serum blocking. Then, the target antibody, HRP enzyme-labeled secondary antibody, and TSA reagent were added successively for antigen repair and serum blocking. After the addition of the target antibody, DAPI was added for nuclear staining. The average fluorescence intensity (sum of fluorescence intensity in the region/area in the region) was used for the semi-quantitative analysis of each indicator. Finally, the Spearman's correlation coefficient was used to calculate the correlations among the indicators. Detailed antibody information used in this experiment is shown in Supplementary file 1: Table 3.

### RNA extraction and quantitative real-time polymerase chain reaction (qRT-PCR)

Total RNA was extracted from tissues and cells using the TRIzol reagent (Invitrogen, USA) and the RNApure TissueCell Kit (CWBIO, CHN), and the RNA concentration was determined using a spectrophotometer. RNA samples (1 μg) were reverse-transcribed into cDNA using a reverse transcription kit (Vazyme, CHN). A Super SYBR Green Kit (Vazyme, CHN, USA) was used to perform qRT-PCR. Each sample was replicated at least three times, and the average value was used for data analysis. The primer sequences for these genes are listed in Supplementary file 1: Table 4.

### Co-culture system

For co-culturing CRC cells and CAFs, after intervention with CAFs, different concentrations of CM supplemented with FBS were collected to culture CRC cells according to the experimental requirements. The cells were cultured in 6-well plates (BIOFIL, 0.4 um, CHN).

### Colony formation assay

Colony formation assay was used to detect the effect of MYL9 knockdown in CAF on tumor cell proliferation. After intervention with CAF (siMYL9-1, siMYL9-2, and siMYL9-NC), the respective CM was collected and inoculated with LoVo and SW480 cell lines into 6-well plates with 800 cells/well. After 10–14 days of culture, the colonies were fixed, stained, and photographed. Three repeated experiments were performed for all cell line groups and the average values were used for comparative analysis.

### Cell counting kit-8 (CCK8) cell proliferation assay

LoVo and SW480 cell lines were treated with complete medium containing different concentrations of the recombinant protein, and five replicates were used for each group. CCK8 solution (10 μL) were added in each well, resulting in 2000 cells. Cells were incubated for 37℃, 3 h, and the absorbance was measured at 450 nm using an enzyme marker. Data were monitored continuously for 5 days and processed using GraphPad Prism V8.0 software (GraphPad Software Inc., California, USA).

### Wound healing assay

LoVo and SW480 cells were seeded into 6-well culture plates, and wounds were made with 200 μL sterile micropipettes when the cells reached 90% confluency. The damaged monolayer cells were washed three times with phosphate buffer solution to remove cell debris. The gap between the two wound edges was measured after incubation with CM for 0 and 24 h. The migration rate was calculated using the following formula: (initial area-final area)/initial area.

### Transwell assay

LoVo and SW480 cells were cultured into the 24-well transwell plates with 8.0 µm pores (Corning Costar, USA) with/without precoated Matrigel (BD, USA; diluted 1:1). CRC cells (LoVo and SW480) (3 × 10^4^) were implanted into the top compartment. After 24 h of culture, the membranes were collected and stained with a crystal violet solution (Solarbio, China). A cotton swab was used to remove cells that did not migrate or invade through the pores. The migrating and invading cells were counted and photographed under a microscope in five different fields.

### Flow cytometry analysis

THP-1 cells were subjected to phorbol ester induced cell adhesion, CAFs were co-cultured according to different groups, and THP-1 cells were blown down and collected in 1.5 mL EP tubes (100 uL), with approximately 1 × 10^6^ cells/well. Antibody (1 ug) TruStain FcX™ (anti-mouse CD16/32) was incubated on ice for 5–10 min, followed by corresponding flow antibody of the target molecule. Zombie Aqua™ Fixable Viability Kit was used for incubation at 37℃ in dark for 45 min. Then, the cells were washed with 1 mL of 1% serum PBS, centrifuged at 2000 rpm for 5 min, and the superserum was discarded. The cell precipitation was again washed twice, and was transferred into the flow tube with 200 uL of 1% serum PBS, and analyzed using BD FACSCanto II flow cytometry. The data were analyzed using FlowJo (V10). The flow cytometric antibodies used in this study were PE anti-human CD163 antibody (Cat# 333606) and APC anti-human CD80 antibody (Cat# 375404).

### Enzyme-linked immunosorbent assay (ELISA)

CM was collected from CAFs, and the concentrations of CCL2, TGF-β1, IL-10, and CXCL1 in CAFs were determined using ELISA (ELISA LAB, Wuhan, CHN) according to the manufacturer's instructions. All sample tests were repeated five times. The absorbance (OD value) was determined using enzyme-labeled instrument at 450 nm wavelength, and the concentrations of CCL2, TGF-β1, IL-10, and CXCL1 in the samples were calculated using standard curve.

### High-throughput CUT&Tag

Briefly, 100000 cells were washed twice gently with wash buffer (20 mM HEPES pH 7.5; 150 mM NaCl; 0.5 mM Spermidine; 1 × Protease inhibitor cocktail). 10μL Concanavalin A coated magnetic beads (Bangs Laboratories) were added per sample and incubated at RT for 10min. Remove unbound supernatant and resuspended bead-bound cells with dig wash buffer (20mM HEPES pH 7.5; 150mM NaCl; 0.5mM Spermidine; 1 × Protease inhibitor cocktail; 0.05% Digitonin; 2mM EDTA) and a 1:50 dilution of primary antibody or IgG control antibody (normal rabbit IgG: Millipore cat.no. 12–370) incubated on a roating platform overnight at 4℃. The primary antibody was removed using magnet stand. Sencondary antibody (Anti-Rabbit IgG antibody, Goat monoclonal: Millipore AP132) was diluted 1:100 in dig wash buffer and cells were incubated at RT for 60 min. cells were washed using the magnet stand 2–3 times in dig wash buffer. A 1:100dilution of pA-Tn5 adapter complex was prepared in dig-med buffer (0.01% Digitonin; 20mM HEPES pH 7.5; 300mM NaCl; 0.5mM Spermidine; 1 × Protease inhibitor cocktail) and incubated with cells at RT for 1h. Cells were washed 2–3 × for 5 min in 1 mL Dig-med buffer. Then cells were resuspended in tagmentation buffer (10mM MgCl2 in Dig-med Buffer) and incubated at 37 °C for 1 h. DNA was purified using phenol–chloroform-isoamyl alcohol extraction and ethanol precipitation. To amplify libraries, 21μL DNA was mixed with 2μL of a universal i5 and auniquely barcoded i7 primer. A volume of 25 μL NEBNext HiFi 2 × PCR Master mix was added and mixed. The sample was placed in a Thermocycler with a heated lid using the following cycling conditions: 72 °C for 5 min (gap filling); 98 °C for 30 s; 14 cycles of 98 °C for 10 s and 63 °C for 30 s; final extension at 72 °C for 1 min and hold at 8 °C. libraried clean-up was performed XP beads (Beckman Counter).

### Dual-luciferase reporter assay and chromatin immunoprecipitation (ChIP)-qPCR

The promoters pGL4.31-hMYL9 promoter-Luc2P-SV40-hRluc and pcDNA3.1-hZEB1 were constructed and verified by DNA sequencing. The indicated plasmid was transfected into cells using Lipofectamine 2000. The cells were collected and tested using a dual-luciferase reporter assay kit (Beyotime, China) 48 h after transfection according to the manufacturer's instructions. ChIP was performed according to the manufacturer's instructions using the ChIP-IT Express Kit, ChIP-IT Express Shearing Kit, and ChIP-IT Protein G Magnetic Beads (ActiveMotif, cat# 53008, 53032, 53014). The primers used for ChIP-qPCR are listed in Supplementary file 1: Table 5.

### Xenotransplantation and metastasis model

Thirty-three BALB/c mice were purchased, of which 18 were used for subcutaneous tumor model construction and 15 were used for lung metastasis model construction. Mice in the two models were randomly divided into three groups: CAFs + LoVo, LoVo, and shMYL9 CAFs + LoVo; the ratio of CAFs to LoVo cells was 1:1. In the subcutaneous tumor and lung metastasis models, LoVo cells were injected into the right inguinal subcutaneous and tail veins of mice, respectively, with a cell volume of 3 × 10^6^. The tumor volume of the subcutaneous tumor model was recorded every three days after tumor formation and calculated using the formula (L × W^2^)/2. The subcutaneous tumor was removed approximately 3–4 weeks after tumor formation. Lung tissue of mice with lung metastasis was removed approximately one month later, and hematoxylin–eosin (HE) staining was performed.

### Bioinformatics analysis

The target genes were identified by three common single-cell sequencing cohorts (GSE132465, GSE144735, Zhou et al. [13]). In the study of clinicopathological characteristics, from The Cancer Genome Atlas (TCGA) (https://portal.gdc.cancer.gov/) and Gene Expression Omnibus (GEO) (https://www.ncbi.nlm.nih.gov/geo/) to download and merge the CRC gene expression and clinical data file and standardizing. TCGA datas was used as training cohort (*n* = 426), Merging multiple GEO data sets (GSE29621, GSE38832, GSE40967, GSE103479) is used as first validation cohort. We previously established transcriptome sequencing data from 150 pairs of CRC patients as a second validation cohort. The overall survival (OS), disease-free survival (DFS), disease-specific survival (DSS), relapse-free survival (RFS) and progression-free survival (PFS) of CRC was analyzed by Xena (https://xena.ucsc.edu/), PrognoScan (http://www.abren.net/PrognoScan/) GEPIA (http://gepia.cancer-pku.cn/) database and GSE40967 and GSE38832 cohort.

Using the “ESTIMATE” and “CIBERSORT” R packages and TIMER database (https://timer.cistrome.org/) MYL9 expression and immune cells infiltrating the correlation analysis of CRC patients. Differential expression of immune checkpoints was verified by analysis in both cohorts. Immunophenoscore (IPS), tumor mutational burden (TMB), and microsatellite instability (MSI) data of CRC patients were obtained from TCIA (https://tcia.at/home), and IPS score was used to predict the response of MYL9 expression to immunotherapy. In addition, relevant immunotherapy research cohort (GSE78220, IMvigor210, GSE19423, Miao et al. [14]) was used to predict MYL9 expression and response to immunotherapy. Use of online tools for pan-cancer analysis, analysis of MYL9 expression and three kinds of the relationship between the three kinds of immunomodulators (http://cis.hku.hk/TISIDB/). The gene ontology (GO) and Kyoto Gene Genome Encyclopedia (KEGG) path analysis were performed after expression grouping by R packaging-clustering analyzer MYL9, and the cut-off value FDR < 0.05. MYL9 expression and drug sensitivity were analyzed in CellMiner database (https://discover.nci.nih.gov/cellminer/).

### Statistical analysis

In this study, low and high MYL9 expression groups were established according to the median expression values of MYL9 mRNA in different data sets. OS, PFS, DFS and DSS were analyzed using Kaplan Meier analysis. Pearson’s or Spearman’s correlation analysis was used. The strength of the correlation was determined using the following guide for the absolute value: 0.00–0.30 (Weak), 0.30–0.70 (Moderate), 0.70–0.90 (strong), 0.90–1.00 (perfect) [15]. R software (4.1.0) was used for all statistical analyses, and the statistical significance was as follows: ns, not significant; * *p* < 0. 05; ** *p* < 0. 01; *** *p* < 0.001; ***** *p* < 0.0001.

## Results

### High expression of MYL9 on CAFs showed a worse prognosis and clinicopathological characteristics of CRC

**Fig. 1:**
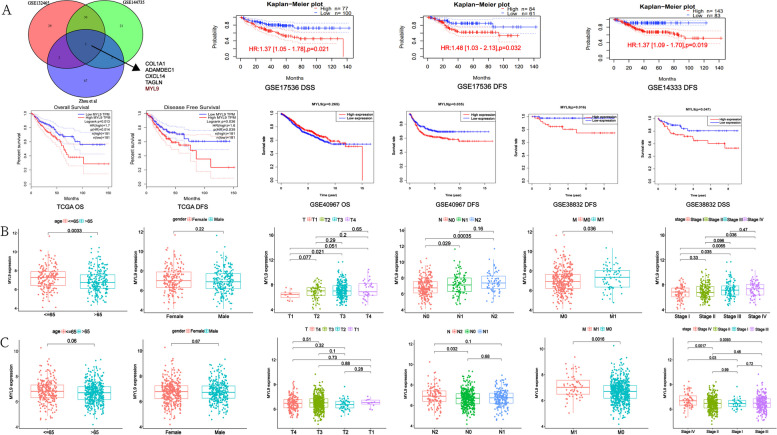
Screening and clinicopathological characteristics of MYL9. **A** Three single-cell sequencing data showed that MYL9 was highly expressed in CAFs and was associated with poor prognosis in CRC patients with OS, DFS, and DSS. **B**-**C** TCGA (**B**) and GEO (**C**) cohorts showed that the high MYL9 expression was closely related to N stage, M stage, and TNM stage in CRC patients. CRC, colorectal cancer; CAFs, cancer-associated fibroblasts; OS, overall survival; DFS, disease-free survival; DSS, disease-specific survival

Three single-cell sequencing cohorts (GSE132465, GSE144735, Zhou et al. [13]) were used for differential gene analysis between CAFs and normal fibroblasts, and the results showed that COL1A1, ADAMDEC1, CXCL14, TAGLN, and MYL9 expression was increased in CAFs. Among these, MYL9 was closely associated with CRC prognosis. The expression of MYL9 and the OS, disease-free survival (DFS), disease-specific survival (DSS), relapse-free survival (RFS), and progression-free survival (PFS) of patients with CRC were analyzed to evaluate the prognostic potential of MYL9. According to the GEPIA database, high MYL9 expression was associated with worse OS and DFS (*p* = 0.013 and *p* = 0.039, respectively). In the PrognoScan database, we also found a strong association between MYL9 overexpression and CRC OS and DFS (GSE17536, OS hazard ratio [HR] = 1.26, 95% confidence interval [CI] 1.00–1.60, *p* = 0.0208; DFS HR = 1.37, 95% CI 1.05–1.78, *p* = 0.032; GSE14333, DFS HR = 1.37, 95% CI 1.09–1.70, *p* = 0.00573). Additionally, two cohorts, GSE38832 and GSE40967, were included in the prognostic analysis. In the GSE38832 cohort, high MYL9 expression had worse DFS and DSS (p = 0.016 and *p* = 0.047, respectively). In the GSE40967 cohort, although high MYL9 expression was not significantly associated with OS, high MYL9 expression was associated with worse RFS (*p* = 0.265 and *p* = 0.035, respectively). These results suggest that active transcription of MYL9 may lead to health risks, and that MYL9 may be a potential prognostic biomarker in patients with CRC (Fig. 1A).

To investigate the relationship between MYL9 mRNA expression and the clinicopathological characteristics of patients with CRC, TCGA and GEO databases (GSE29621, GSE38832, GSE40967, and GSE103479) were analyzed. In the TCGA cohort, high expression of MYL9 was closely related to advanced age, N stage, M stage, and TNM stage of CRC (Fig. 1B). In the GEO cohort, we found similar results to those in the TCGA cohort after combining multiple datasets (Fig. 1C). The results of the two study cohorts suggested that the expression of MYL9 in patients with N2 and M1 stage was higher than that in patients with N0 and M0 stage, and the expression of MYL9 in patients with Stage IV, Stage III CRC was significantly higher than that in patients with Stage I, Stage II CRC, suggesting that the expression of MYL9 was closely related to the development and metastasis of CRC.

We also analyzed the prognostic value of MYL9 expression in 31 non-CRC tumors. We found that high MYL9 expression in adrenocortical carcinoma, brain lower-grade glioma, and mesothelioma was associated with poor OS. High expression of MYL9 in ovarian serous cystadenocarcinoma indicated worse DFS; MYL9 in adrenal cortical carcinoma, prostate adenocarcinoma, and kidney renal clear cell carcinoma showed high DSS expression, and RFS was worse (Figure S1A). In the analysis of clinicopathological characteristics, MYL9 expression was closely related to the tumor TNM stage in bladder urothelial carcinoma, adrenocortical carcinoma, stomach adenocarcinoma, and thyroid carcinoma (Figure S1B). In addition, MYL9 was differentially expressed in many cancer types such as stomach adenocarcinoma, prostate adenocarcinoma, invasive carcinoma, liver hepatocellular carcinoma, lung adenocarcinoma, and squamous cell carcinoma. Subsequently, we analyzed the relationship between MYL9 and tumor mutation burden (TMB) and microsatellite instability (MSI) in these tumors. The results showed that MYL9 expression was closely associated with TMB and MSI in stomach adenocarcinoma, skin cutaneous melanoma, and lung squamous cell carcinoma (Figure S1C). Considering that whether MYL9 is an important immunotherapy target in tumors requires further study, we performed a pan-cancer analysis of MYL9 expression and immunomodulators. Our results showed that MYL9 expression was positively correlated with multiple immunoinhibitor, immunostimulators, and major histocompatibility complex molecules in various tumors (Figure S1D). Finally, we performed a drug sensitivity analysis of MYL9 and found that patients with high MYL9 expression were highly sensitive to receptor tyrosine kinase inhibitors (Figure S1E). Therefore, MYL9 expression is closely related to the prognosis of various tumors and tumor immunity, and may be a potential target for tumor prognosis and immunotherapy.

### MYL9 was localized to CAFs and total protein expression was elevated in CRC

**Fig. 2:**
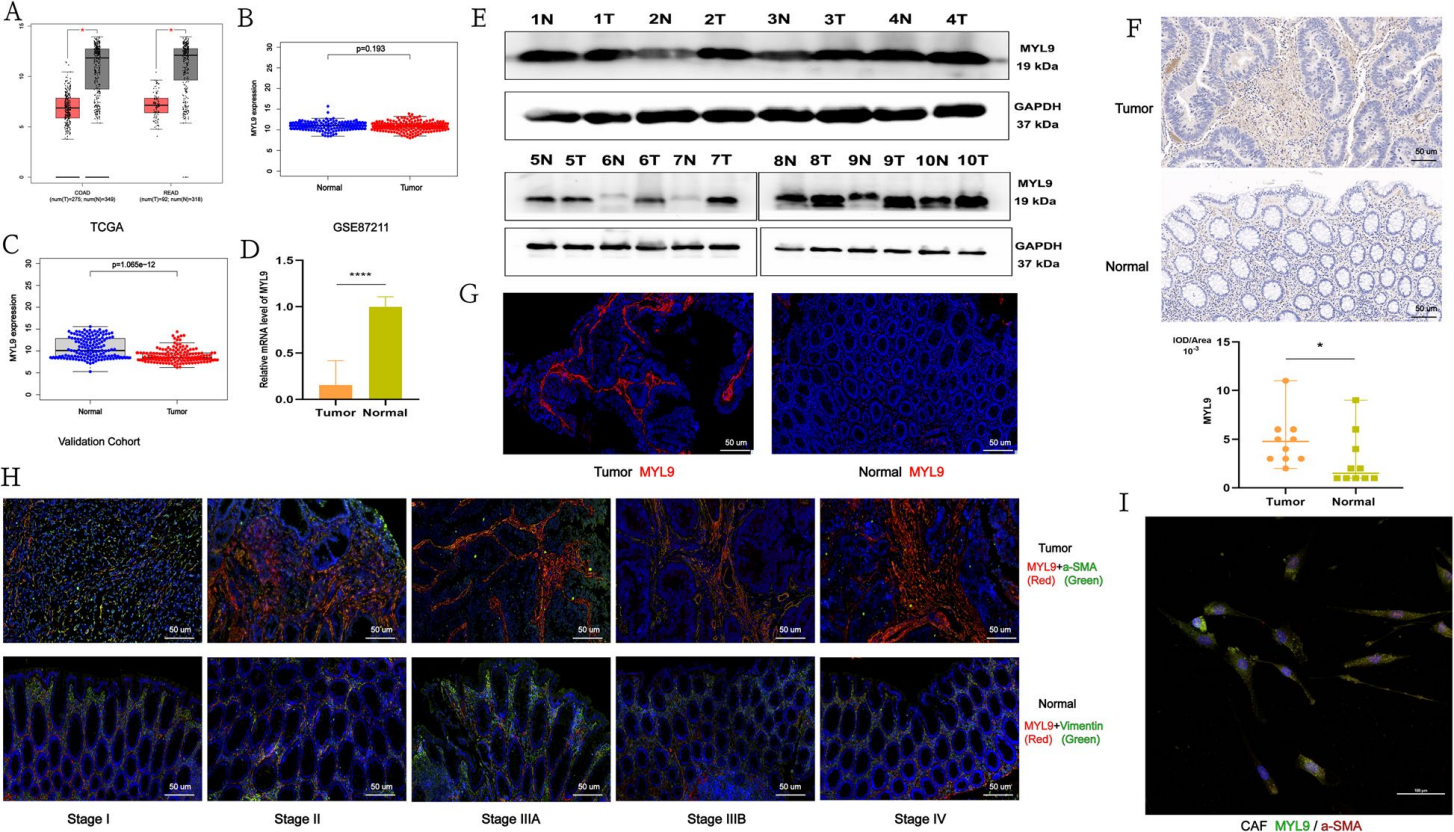
The expression level of MYL9 gene in CRC and cell localization. **A**-**C** The expression levels of MYL9 gene in CRC and normal tissues were determined using TCGA cohort (**A**), GSE87211 cohort (**B**), and validation cohort (**C**). **D** In 30 pairs of fresh CRC and normal tissues, low MYL9 expression was found in CRC by qRT-PCR. **E** The level of MYL9 protein in CRC was higher than that in normal tissues. **F** IHC found that MYL9 was highly expressed in CRC, mainly in tumor stroma (Scale bar = 50μm). **G** Tissue immunofluorescence indicated that MYL9 protein was highly expressed in tumor, mainly in tumor stroma (Scale bar = 50μm, Red: MYL9). **H** Tissue immunofluorescence found that MYL9 was mainly located in CAFs in CRC, and with the increase in TNM stage, the expression level of MYL9 protein and CAFs cells also increased (Scale bar = 100μm, Red: MYL9; Green: a-SMA). **I** Immunofluorescence of primary CAFs colocalized with MYL9 (Scale bar = 100μm, Red: a-SMA; Green: MYL9). CRC, colorectal cancer; CAFs, cancer-associated fibroblasts; IHC, immunohistochemistry

The level of MYL9 expression in CRC and its localization is still controversial. Studies have found that low MYL9 expression is associated with poor prognosis in CRC [16], but Zhao et al. [17 ] found that MYL9 is highly expressed in early onset CRC. In addition, Feng et al. [18] studied the expression of MYL9 in CRC cells and found that it promoted tumor progression. Therefore, the localization and expression levels of MYL9 in CRC require further verification. To determine MYL9 localization and expression levels, validation was performed using multiple cohorts. The GEPIA database was used to analyze MYL9 mRNA expression levels in colon cancer, rectal cancer, and normal tissues. The results showed that MYL9 mRNA expression was lower in colon and rectal cancers than in normal tissues (Fig. 2A). In the GSE87211 cohort, there was no significant difference in MYL9 mRNA expression between tumor and normal tissues (Fig. 2B). In our 150 pairs of CRC transcriptome data, we found that MYL9 mRNA expression was lower in tumors than in normal tissues (Fig. 2C). In addition, qRT-PCR analysis of 30 pairs of fresh CRC tissues showed similar results (Fig. 2D).

In terms of protein level, western blotting and IHC results of 10 pairs of fresh CRC samples indicated that the total MYL9 protein level in tumor tissues was higher than that in normal tissues (Fig. 2E, F). MYL9 mRNA expression level is inconsistent with protein expression level, which may be caused by post-transcriptional or post-translational regulation, which still needs to be verified by later experiments. Interestingly, the IHC results showed that MYL9 was mainly expressed in the tumor stroma (Fig. 2F). Subsequently, we conducted tissue immunofluorescence verification, and the immunofluorescence results indicated that the protein expression level of MYL9 was higher in tumors than in normal tissues and was mainly concentrated in the tumor stroma (Fig. 2G). In order to clarify the location of MYL9, analysis of single-cell sequencing data (GSE132465, GSE144735) showed that MYL9 was mainly expressed in tissue stem cells and fibroblasts, and it was less expressed in epithelial cells (Figure S2A). Therefore, we hypothesized that MYL9 plays a role in CAFs to influence CRC progression. Using TIMER 2.0 database, we found that the expression of MYL9 was highly correlated with CAFs infiltration in colon and rectal cancers (COAD, *r* > 0.8, *p* < 0.0001; READ, *r* > 0.6, *p* < 0.001) (Figure S2B). In addition, tissue immunofluorescence showed that MYL9 and the CAFs marker protein a-SMA were co-expressed in the tumor stroma. With an increase in the CRC TNM stage, the expression levels of MYL9 and CAFs also increased, whereas the expression levels of MYL9 were low in normal tissues (Fig. 2H). Finally, we successfully isolated primary CAFs from four CRC patients and cellular immunofluorescence also confirmed that MYL9 was expressed in CAFs (Fig. 2I, Figure S2C, D). After identification and culture of primary CAFs, stably proliferating primary CAFs were regarded as CAFs cell lines for further study. Tissue immunofluorescence showed that MYL9 was stably expressed in CAFs rather than in tumor cells, considering that the primary CAFs that we isolated were also stably cultured and passaged. Therefore, we intended to verify the expression of MYL9 in CAFs, tumor cell lines (LoVo, SW480, and HCT116), and normal cell lines (NCM460) by western blotting and found that MYL9 was stably expressed in CAFs and was less expressed in CRC and normal cell lines (Figure S2E). Therefore, we found that MYL9 was mainly localized to CAFs rather than to tumor epithelial cells and that its protein expression was elevated in CRC.

### Silencing MYL9 in CAFs inhibits the proliferation, migration, and invasion of CRC cells in vitro

**Fig. 3:**
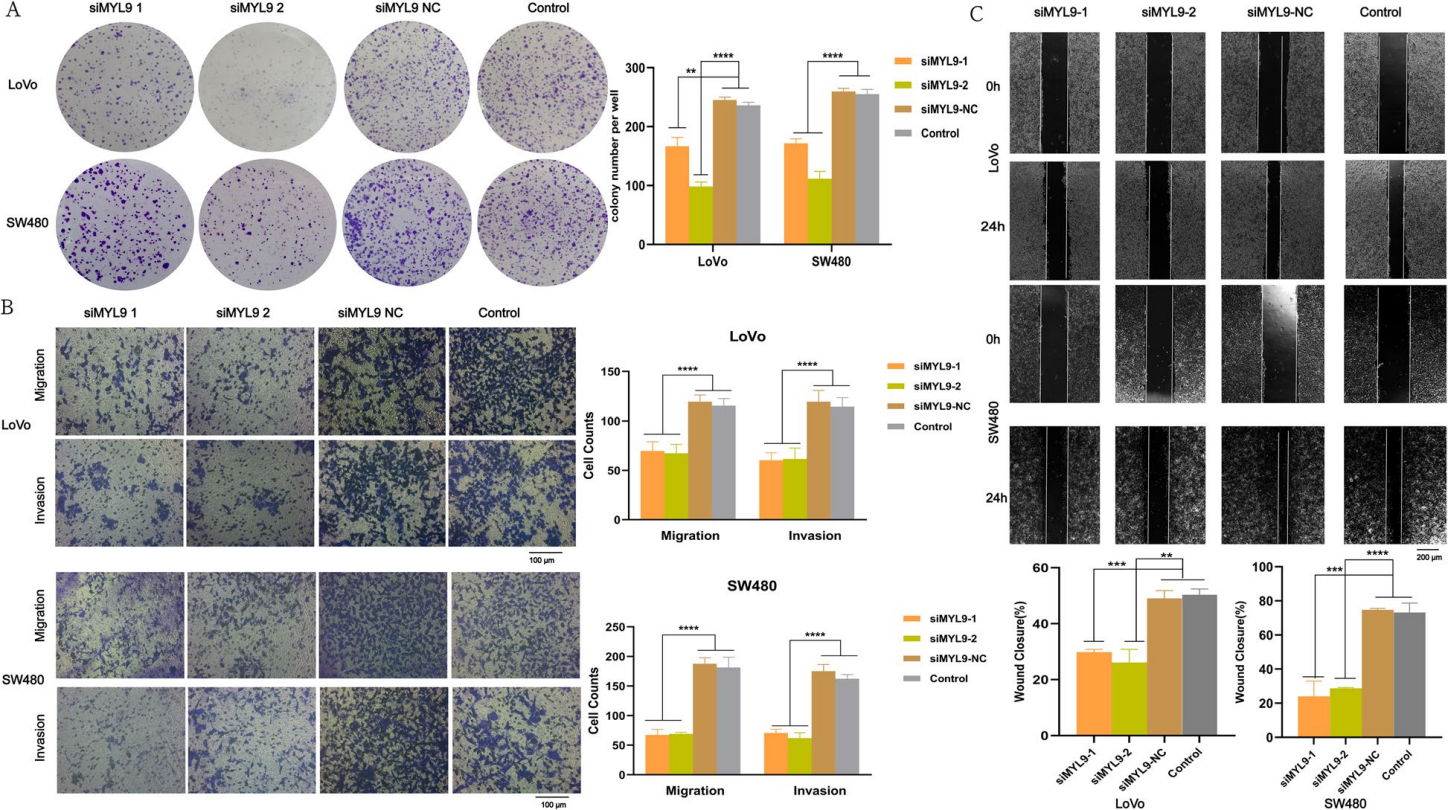
Silencing MYL9 in CAFs inhibited the proliferation, migration, and invasion of LoVo and SW480 cells. **A** Colony formation assays on LoVo and SW480 cells suggested that MYL9 after silencing could inhibit CRC proliferation. **B**-**C** Transwell (**B**) and wound healing (**C**) assays showed that MYL9 silencing inhibited the migration and invasion ability of LoVo and SW480 cells. Transwell Scale Bar = 100μm; Wound healing Scale Bar = 200μm. Each bar represents the mean ± SD of the three independent experiments. CRC, colorectal cancer; CAFs, cancer-associated fibroblasts; SD, standard deviation

Given that CAFs play an important role in CRC progression, we hypothesized that MYL9 silencing could inhibit the effects of CAFs on CRC. To verify this, we collected CM from primary CAFs silenced MYL9 with siRNA and co-cultured them with LoVo and SW480 cells to detect their biological functions. Colony formation assay showed that silencing MYL9 significantly inhibited the proliferation ability of CRC cells (Fig. 3A, Figure S2F). Transwell assay showed that the migration and invasion abilities of CRC cells were significantly inhibited when MYL9 was silenced in CAFs (Fig. 3B). In addition, wound-healing assay indicated that MYL9 silencing inhibited CRC cell migration (Fig. 3C). These results confirm that high MYL9 expression in CAFs may be key to the proliferation, migration, and invasion of CRC cells in vitro.

### High expression of MYL9 was associated with high M2 macrophages infiltration

Tumor and TME are closely related and often referred to as the relationship between “seed” and “soil.” We scored the CRC TME in TCGA database and performed a correlation analysis with MYL9. The results showed that the expression of CRC and MYL9 had a moderately strong correlation with the tumor immune and stromal scores, suggesting that MYL9 may play an important role in the TME (Figure S3A). Immune cell infiltration is associated with tumor prognosis, and although some biological information analysis indicated that MYL9 has an effect on CRC tumor immune cell infiltration, there is a lack of data verification. Therefore, we used TCGA, GEO databases, and our own CRC transcriptome data as validation cohorts to analyze the differences and correlations between MYL9 expression and immune cell infiltration. In the TCGA cohort, we found that MYL9 expression showed significant differences and a high correlation with memory B cell, CD4 memory resting T cells, helper T cells, regulatory T cells (Tregs), gamma delta T cells, monocyte, macrophages M0, macrophages M1, macrophages M2, and activated dendritic cell infiltration (Figure S3B). Similarly, we found that MYL9 expression showed significant differences and a strong correlation with plasma cells, CD4 memory resting T cells, resting NK cell, monocyte, macrophages M0, macrophages M1, macrophages M2, activated dendritic cells, resting mast cells, and neutrophils cells infiltration in the GEO validation cohort (Figure S3C). In addition, using our own transcriptome sequencing data as a second validation cohort, we also found that MYL9 expression is associated with plasma cells, CD4 memory resting T cells, CD4 memory activated T cells, macrophages M2, activated dendritic cells infiltration, showing significant differences and high correlation (Figure S3D). We conducted a comprehensive analysis of the results from the three cohorts and found that high MYL9 expression in CRC was closely associated with low CD4 memory resting T cells, activated dendritic cell, and high M2 macrophage infiltration.

TIMER and GEPIA databases were used to investigate the relationship between MYL9 expression and various immune cell marker genes in colon and rectal cancers. The results showed that MYL9 expression levels significantly correlated with the most significant marker genes of various immune cells in colon and rectal cancers, including M2 macrophages, tumor-associated macrophages (TAMs), and dendritic cells (Supplementary file 1: Table 6).

**Fig. 4:**
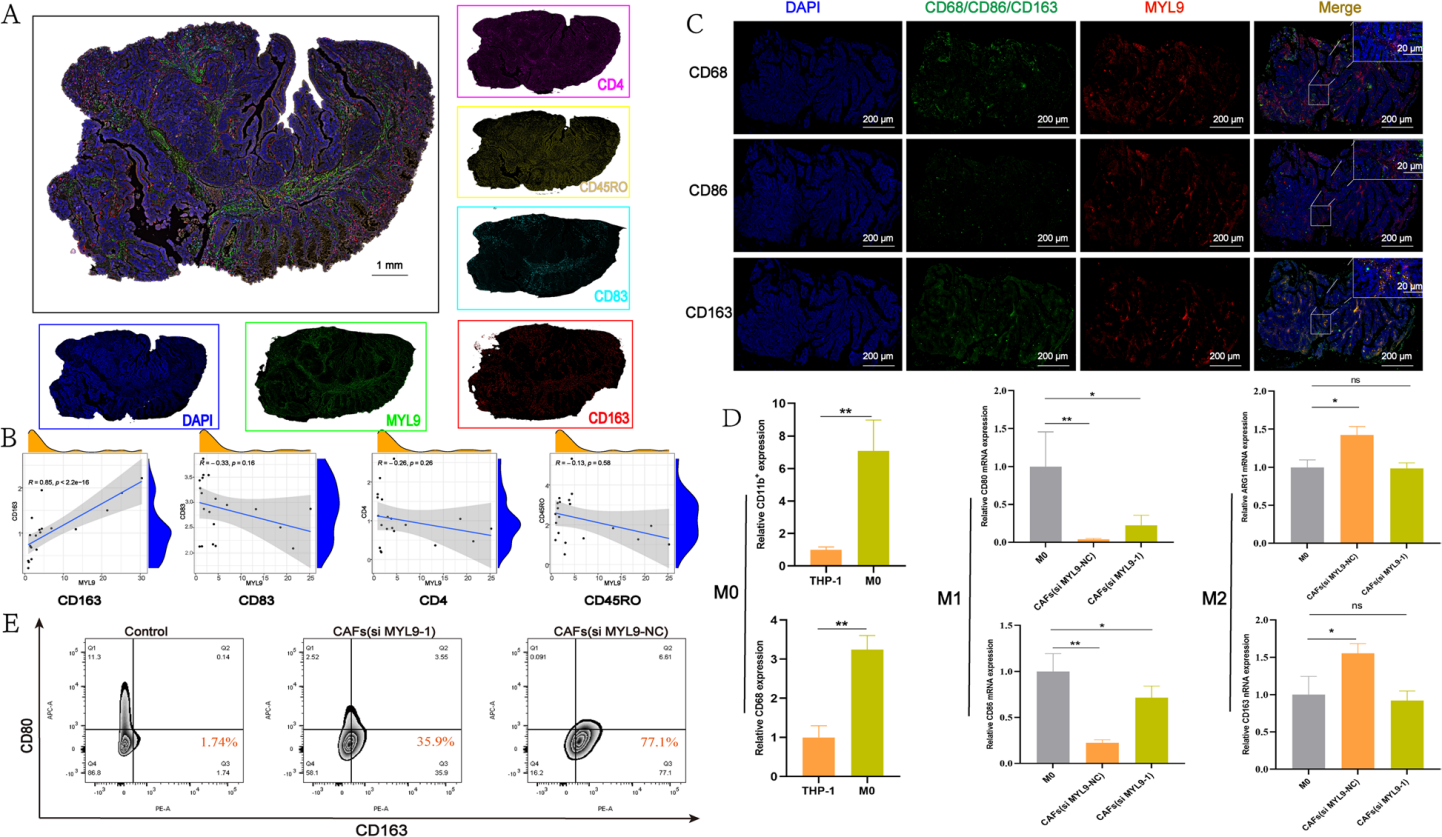
High expression of MYL9 was associated with CRC immunosuppressive microenvironment. **A** Slides were simultaneously stained with a multiplex immunofluorescent panel containing CD163 (red), MYL9 (green), CD83 (cyan), CD4 (pink), CD45RO (yellow), and DAPI (blue); Scale Bar = 1000μm. **B** Spearman’s correlation coefficient evaluated the correlation between MYL9 and CD4 memory resting T cells (CD4, CD45RO), activated dendritic cells (CD83), and M2 macrophage infiltration (CD163), and found that the expression of MYL9 is closely related to M2 macrophage infiltration. **C** Tissue immunofluorescence results indicated that the expression of MYL9 in CRC tissue was strongly correlated with M2 macrophage infiltration. **D** PCR results suggested that the polarization of M0 macrophages to M2-type macrophages was significantly attenuated by silencing MYL9 in CAFs. **E** Cytometry also confirmed that M0 macrophages polarized towards M2 macrophages after silencing MYL9. CRC, colorectal cancer; PCR, polymerase chain reaction; CAFs, cancer-associated fibroblasts

Finally, we performed multiple immunofluorescence assays on 20 paraffin-embedded samples of CRC to verify the relationship between MYL9 and CD4 memory resting T cells, activated dendritic cells, and M2 macrophages. CD163 antibody-labeled M2 macrophages, CD83 antibody-labeled activated dendritic cells, and CD4 and CD45RO antibody-labeled CD4 memory resting T cells were used. Our results showed that there was a significant positive correlation between MYL9 and CD163 expression (*R* = 0.85, *p* < 0.001), MYL9 was negatively correlated with CD83 expression (*R* = -0.33, *p* = 0.16), and the expression of CD4 and CD45RO was negatively correlated with MYL9 expression (*R* = -0.26, *p* = 0.26; *R* = -0.13, *p* = 0.58, respectively) (Fig. 4A, B). Therefore, the expression of MYL9 is closely associated with M2 macrophage infiltration.

Considering the abundance of TAM in the tumor, TME has mixed macrophage populations, and changes in TAM can also affect the balance between the pro-tumor and anti-tumor activities of monocytes. We further analyzed the relationship between the expression of MYL9 and TAM. The relationship between MYL9 expression and M0, M1, and M2 macrophage infiltration was determined by immunofluorescence and IHC using continuous sections of paraffin-embedded CRC specimens. Tissue immunofluorescence results indicated that MYL9 expression was closely correlated with M2 macrophage infiltration but weakly correlated with M0 and M1 macrophage infiltration (Fig. 4C). Similar results were obtained by IHC. In the area with high MYL9 expression, the positive proportion of M2 and M0 macrophages was significantly higher than that of M1 cells (Figure S3E). Subsequently, after THP-1 cells were induced to differentiate into M0 macrophages with phorbol-12-myristate-13-acetate, CAFs with or without MYL9 silencing were co-cultured to determine whether MYL9 could induce M0 cells to differentiate into M1 or M2 macrophages, and PCR results showed that after MYL9 silencing, M0 differentiation into M2 macrophages was significantly attenuated compared with normal CAFs (Fig. 4D). Flow cytometry also showed that the percentage of M2 cells decreased significantly after MYL9 silencing compared to that in the normal CAFs group (Fig. 4E). Therefore, we believe that MY9 located in CAFs can promote the polarization of M0 to M2 macrophages.

### MYL9 expression may predict the clinical immunotherapy efficacy of CRC

**Fig. 5:**
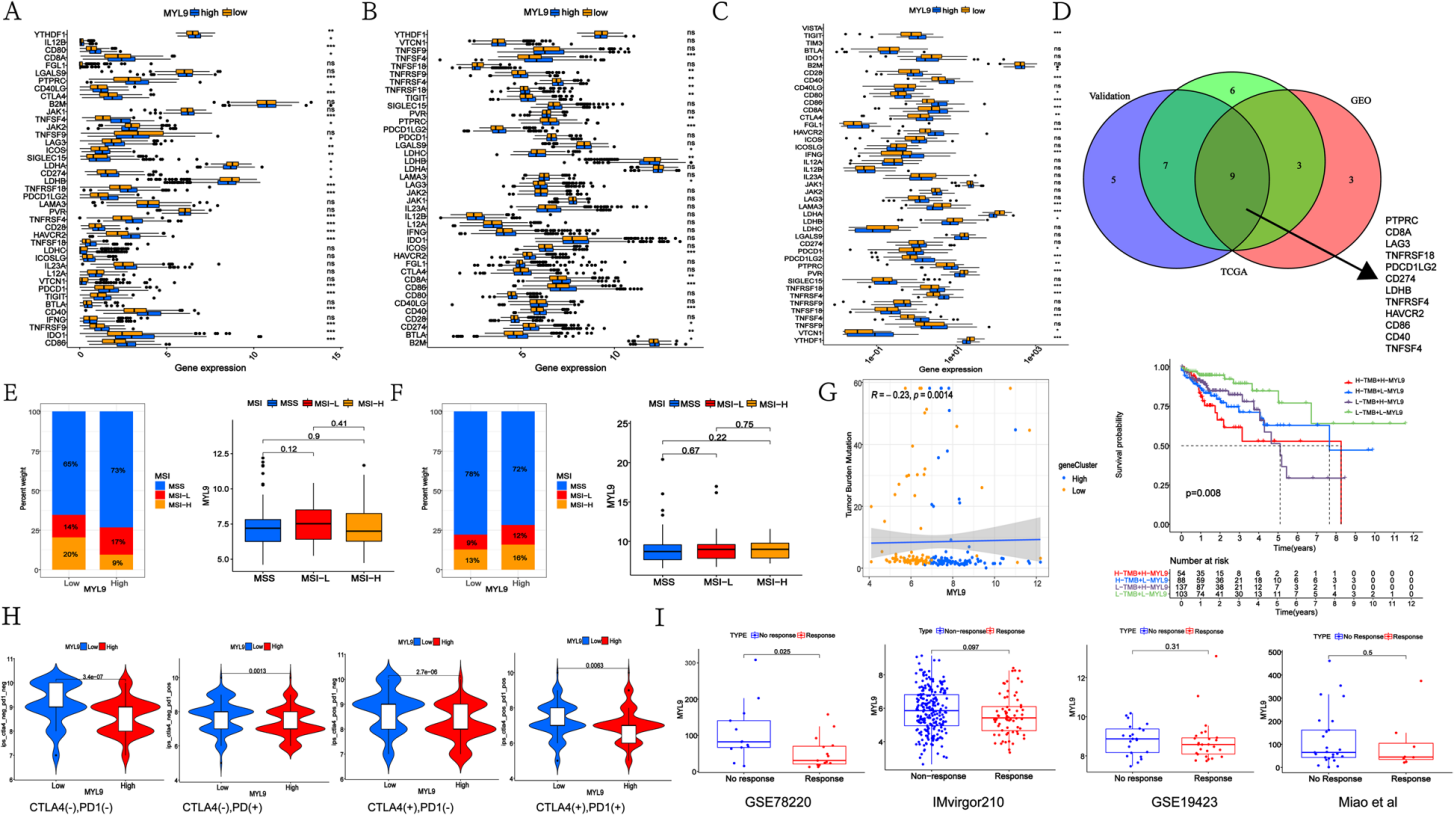
The high MYL9 expression was not sensitive to tumor immunotherapy. A-**C** Three cohorts analysis of the difference between MYL9 expression and common immune checkpoint expression in three cohorts. **A** TCGA cohort, **B** GEO cohort, **C** Validation cohort from our own transcriptome data. **D** Intersection analysis of three study cohorts. E–F: TCGA (**E**) and GSE24551 (**F**) cohort differential analysis of MYL9 expression and CRC MSS, MSI-L, and MSI-H. **G** Correlation and survival analyses between MYL9 and TMB. Low TMB and high MYL9 expression had worse prognosis. **H** MYL9 expression and IPS showed that CRC with high MYL9 expression has lower immunogenicity. **I** Analysis of MYL9 expression and tumor immunotherapy reactivity in four study cohorts showed that the tumors with high MYL9 expression were less responsive to immunotherapy. CRC, colorectal cancer; MSS, microsatellite stability; MSI, microsatellite instability; TMB, tumor mutation burden; IPS, Immunophenoscore

Since MYL9 is mainly expressed in CAFs, to further clarify the role of MYL9 in immunotherapy, we divided the TCGA, GEO, and our validation cohorts into high and low groups according to the expression of MYL9. We analyzed the relationship between MYL9 expression and common immune checkpoints. The results of the three cohorts showed that MYL9 expression correlated with PTPRC, CD8A, LAG3, TNFRSF18, PDCD1LG2, CD274, LDHBTNFRSF4, HAVCR2, CD86, CD40, and TNFSF4 expression (Fig. 5A-D). MSI and TMB are considered important molecular markers for predicting the prognosis and immune efficacy of CRC. Therefore, we analyzed whether there was a correlation between TMB, MSI, and MYL9 expression. In our study, no significant differences were found between MYL9 expression and MSS, MSI-L, and MSI-H in TCGA and GSE24551 cohorts (Fig. 5E, F), and there was a negative correlation between TMB and MYL9 expression (*r* = -0.23, *p* = 0.0014). Further studies found that patients with CRC with high MYL9 expression and low TMB had worse OS than those in the other groups (Fig. 5G).

We further investigated whether MYL9 could predict patient response to immune checkpoint blockade therapy. First, we analyzed the expression of MYL9 and CRC IPS. Our results showed that high MYL9 expression was associated with lower IPS, which also suggested that CRC patients with high MYL9 expression had lower immune prototypes and worse immune responses (Fig. 5H). Considering that the expression of MYL9 can affect the response of patients with CRC to immunotherapy, we used GSE78220 (*n* = 28), GSE19423 (*n* = 48), Miao et al. [14] (*n* = 35), and IMvirgor210 (*n* = 298) cohorts to study whether MYL9 expression can predict patients’ response to immunotherapy. In the GSE78220 cohort, higher MYL9 expression was associated with worse immunotherapy response (*p* = 0.025). In the GSE19423, Miao et al. [14], and IMvirgor210 cohorts, although there was no statistically significant difference between the high and low expression of MYL9 and the immunotherapy response, we found that the high expression of MYL9 in these cohorts tended to lead to a poorer immunotherapy response (Fig. 5I). Overall, in analyzing the relationship between MYL9 and immune checkpoints, TMB, and immunotherapy, we found that patients with high MYL9 expression benefited less from immune checkpoint therapy and that MYL9 may be a new target for immunotherapy.

### MYL9 may recruit or regulate M2 macrophage infiltration by regulating CCL2 and TGF-β1 secreted by CAFs

**Fig. 6:**
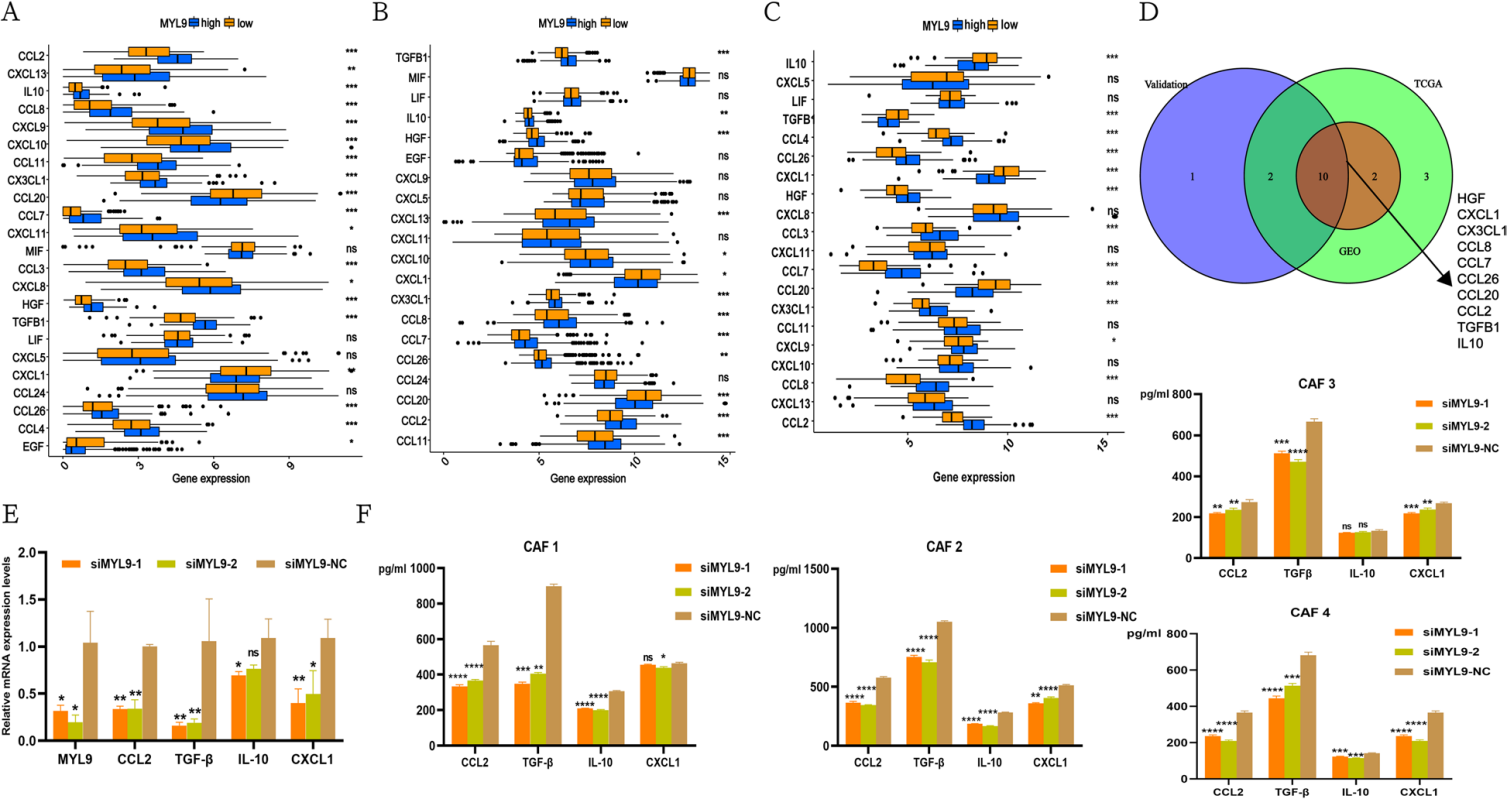
MYL9 silencing can reduce CAFs secretion of CCL2 and TGF-β1. **A**-**C** Analysis of the difference between MYL9 expression and common cytokines and chemokines expression in three cohorts. **A** TCGA cohort, **B** GEO cohort, **C** Validation cohort from our own transcriptome data. D: Intersection analysis of three study cohorts. **E** qRT-PCR showed that MYL9 silencing could significantly reduce the secretion of CCL2 and TGF-β1. **F** ELISA showed that after MYL9 silencing, the levels of CCL2 and TGF-β1 protein expressed by the four primary CAFs were significantly reduced. CAFs, cancer-associated fibroblasts; qRT-PCR, quantitative real-time polymerase chain reaction; ELISA, enzyme-linked immunosorbent assay

CAFs exert multiple effects on cancer cells through direct cell contact or paracrine cytokines. Therefore, we investigated whether MYL9 regulates CAFs cytokine secretion. Similarly, TCGA, GEO, and our validation cohorts were divided into two groups, high and low, according to the expression of MYL9, and a relationship between the expression of MYL9 and some common cytokines and chemokines was observed. In the three cohorts, prompt MYL9 high expression and HGF, CXCL1, CX3CL1, CCL8, CCL7, CCL26, CCL20, CCL2, TGFB1, and IL10 expression were closely related (Fig. 6A-D). After qRT-PCR verification, we found that the expression of CCL2, TGF-β1, IL-10, and CXCL1 decreased after MYL9 was silenced, and the changes in CCL2 and TGF-β1 were particularly significant (Fig. 6E, Figure S4A). Subsequently, we silenced MYL9 in four isolated primary CAFs cases and extracted cell supernatants for ELISA. The results showed that after MYL9 silencing, the protein levels of CCL2, IL-10, CXCL1, and TGF-β1 decreased, and the stable reduction in CCL2 and TGF-β1 was more significant (Fig. 6F). These results strongly suggested that MYL9 regulates the secretion of cytokines and chemokines in CAFs.

A close relationship between CCL2, TGF-β1, and M2 macrophages has been confirmed by previous studies. CCL2 is a chemokine that attracts immune cells to migrate to sites of inflammation. In the TME, the role of CCL2 is particularly obvious; it can attract M2 macrophages to accumulate in tumor tissues. When M2 macrophages are subjected to the chemotaxis of CCL2, they play an immunosuppressive role [19]. In tumor immunotherapy, blocking the CCL2 signaling pathway can effectively reduce the infiltration of M2 macrophages and enhance the antitumor immune response to achieve the purpose of tumor treatment [20]. In addition, TGF-β1 is an important cytokine that plays an important regulatory role in the growth, differentiation, and function of immune cells. TGF-β1 can promote the differentiation and growth of M2 macrophages, and can regulate its function, thereby promoting the growth and invasion of tumors [21, 22]. Therefore, we believe that MYL9 may affect the tumor immune microenvironment by regulating the secretion of CCL2 and TGF-β1 from CAFs, and is closely related to M2 macrophage infiltration. In addition, whether CCL2 and TGF-β1 can promote tumor progression in addition to affecting the tumor immune microenvironment and the regulatory mechanism of MYL9 require further investigation.

### CAFs secretes CCL2 and TGF-β1 and synergistically promote the proliferation, migration, and invasion of CRC

In addition to affecting the TME, whether CCL2 and TGF-β1 can promote CRC independently or synergistically remains unclear. Studies have shown that both CCL2 and TGF-β1 can promote CRC progression [23, 24]. However, whether MYL9 promotes CRC progression through CCL2 or TGF-β1 or both requires further investigation. We combined LoVo and SW480 cells with recombinant proteins CCL2 (20 ng/mL) and TGF-β1 (0.1 ng/ml) to detect their biological functions. The CCK-8 cell proliferation assay result showed that the combination of TGF-β1 and CCL2 significantly improved the proliferation ability of CRC cells compared with the single use (Figure S4B, C). Transwell migration and invasion assays also found that the migration and invasion ability of LoVo and SW480 cells was significantly increased after the combination of CCL2 and TGF-β1 compared with the control, CCL2 and TGF-β1 groups (Figure S4D). Therefore, we found that MYL9 plays a role in CRC progression by regulating the secretion of CCL2 and TGF-β1 in CAFs.

### MYL9 knockdown in CAFs inhibits the proliferation and metastasis of CRC cells in vivo

**Fig. 7:**
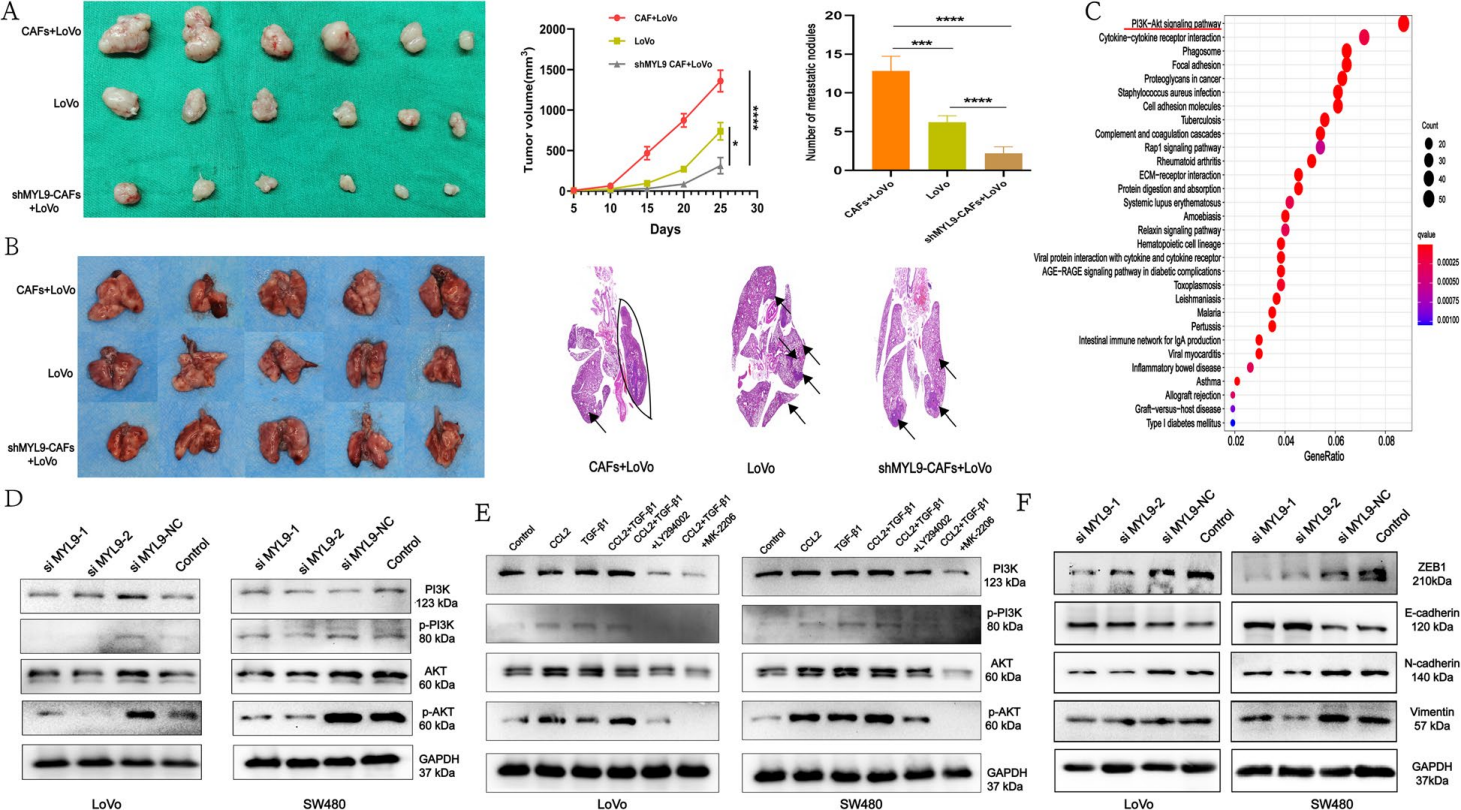
In vivo experiment and mechanism pathway validation. **A**-**B** Subcutaneous tumor and lung metastasis models in nude mice suggest that MYL9 knockdown can inhibit the growth of CRC and lung metastasis. **C** Three cohorts suggest that MYL9 may function through the PI3K-AKT pathway. **D** Western blotting demonstrated that silencing MYL9 decreased the phosphorylation of PI3K and AKT in CRC cells. **E** The synergistic effect of CCL2 and TGF-β1 promotes CRC cell progression through the PI3K-AKT pathway. **F** Silencing MYL9 in CAFs can affect the EMT progression of CRC. CRC, colorectal cancer; CAFs, cancer-associated fibroblasts; EMT, epithelial-mesenchymal transition

To study the biological effect of MYL9 gene silencing in CAFs on CRC, we used a lentivirus to steadily knockdown the MYL9 gene in CAFs, CAFs with or without MYL9 gene knockdown and LoVo cells were injected subcutaneously and intravenously at a ratio of 1:1 to induce tumorigenesis and metastasis in nude mice. After MYL9 was knocked out, the ShMYL9 + LoVo group developed smaller tumors and fewer lung metastatic nodules than the LoVo and CAFs + LoVo groups (Fig. 7A, B, Figure S4E).

### MYL9 may promote CRC progression through the CCL2/TGF-β1/PI3K/AKT axis

In vivo and in vitro studies confirmed that silencing or knockdown of MYL9 can inhibit the proliferation and metastasis of CRC cells. To explore the biological functions and signaling pathways of MYL9, we divided the expression of MYL9 into high and low groups for KEGG and GO enrichment analyses. The signaling pathway between MYL9 and CRC mainly involves the PI3K-AKT pathway (Fig. 7C, Figure S5). To verify this result, after MYL9 was silenced in CAFs, the CM was collected and co-cultured with CRC cells (LoVo and SW480), and protein changes in the PI3K-AKT pathway were observed by western blotting. Our results showed that PI3K, AKT, phosphorylated p-PI3K, and phosphorylated p-AKT proteins increased after co-culture of LoVo cells with CAFs, and phosphorylated p-PI3K and p-AKT protein levels decreased after MYL9 silencing. Similar results were found in SW480 cells, where the phosphorylation of p-PI3K and p-AKT also decreased after MYL9 silencing (Fig. 7D).

We have confirmed that MYL9 can reduce the secretion of CCL2 and TGF-β1 by CAFs, while CCL2 combined with TGF-β1 can promote the proliferation, migration, and invasion of CRC cells. Therefore, to test the hypothesis whether MYL9 act on CRC via the CCL2/TGFβ1/PI3K/AKT axis, CRC cells were treated with recombinant CCL2 (20 ng/mL), TGF-β1 (0.1 ng/mL), and PI3K-AKT pathway inhibitors LY294002 (0.5 ug/mL). Western blotting results showed that CCL2 combined with TGFβ1 could promote the expression of PI3K, phosphorylated p-PI3K, AKT, and phosphorylated p-AKT in LoVo and SW480 cells. After the application of PI3K inhibitor (LY294002), it can block PI3K and AKT phosphorylation in LoVo and SW480 cells stimulated by CCL2 combined with TGF-β1 (Fig. 7E). Therefore, we confirmed that MYL9 in CAFs promotes the proliferation of CRC cells by regulating the secretion of CCL2 and TGF-β1 and through the PI3K-AKT pathway.

### Silencing MYL9 expression in CAFs can inhibit EMT progression of CRC

MYL9 expression in CAFs not only affects the proliferation of CRC cells but also promotes their migration and invasion. Current studies have confirmed that TGF-β is the main inducer of EMT, which can facilitate CRC EMT process [25–27]. Our study found that MYL9 could regulate the secretion of TGF-β1, which had an impact on TME and EMT. Therefore, we speculated that MYL9 could also promote the occurrence of CRC EMT. To verify this hypothesis, CM was extracted after MYL9 was silenced in CAFs using siRNAs and co-cultured with CRC cells. The results showed that after MYL9 silencing, the expression of N-cadherin, vimentin, and the transcription factor ZEB1 decreased and the expression of E-cadherin increased in LoVo and SW480 cells (Fig. 7F). Therefore, we found that MYL9 in CAFs promotes EMT progression in CRC cells.

### MYL9 binding to IQGAP1 through the ERK 1/2 pathway regulates the secretion of CCL2 and TGF-β1 in CAFs

**Fig. 8:**
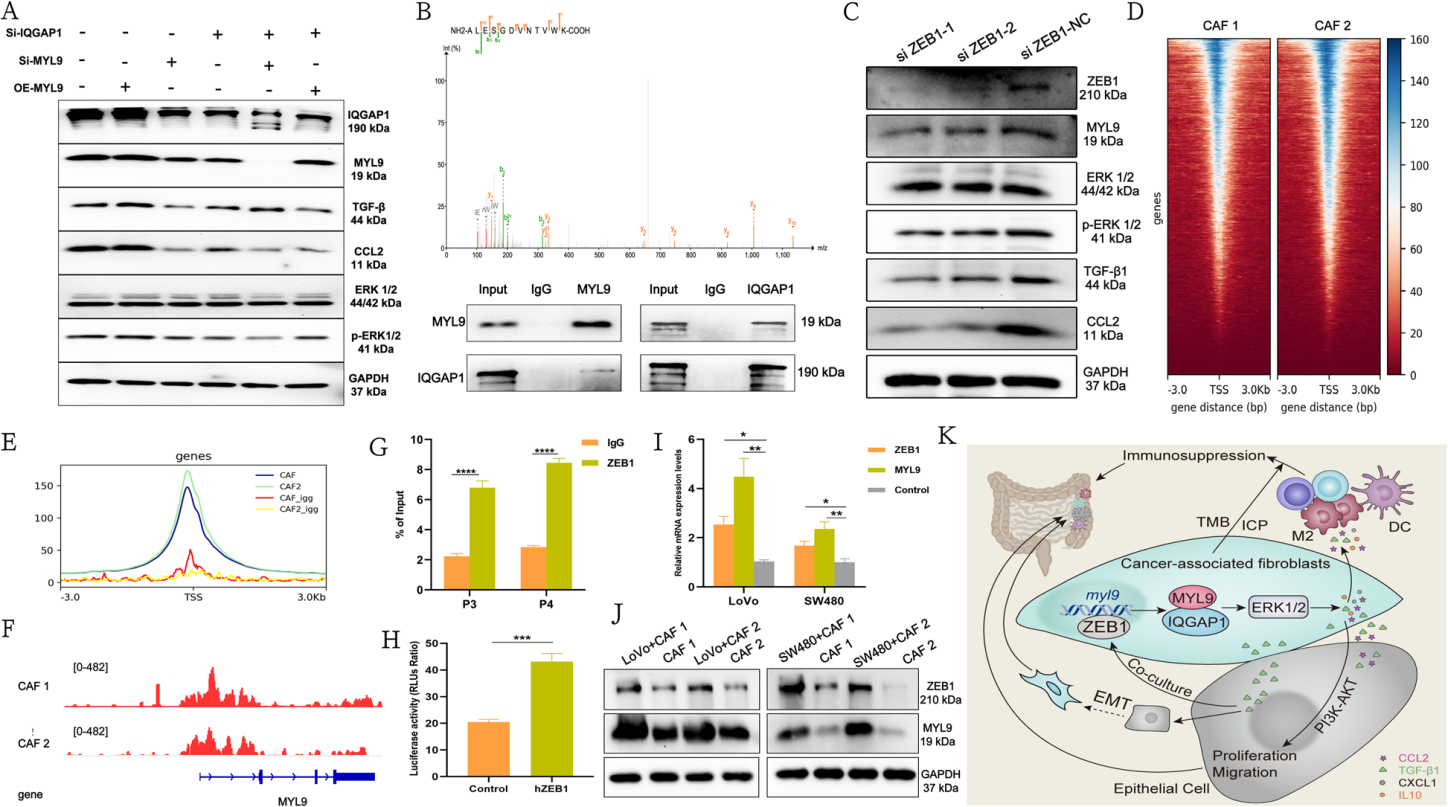
Upstream and downstream regulation mechanism of MYL9 in CAFs. **A** Western blotting showed that MYL9 could bind to IQGAP1 to regulate CCL2 and TGF-β1 secretion through the ERK 1/2 pathway. **B** Protein spectral analysis and Co-IP assay showed MYL9 can interact with IQGAP1 protein. **C** The expression of MYL9, phosphorylated ERK1/2, CCL2, and TGF-β1 decreased with the silencing of ZEB1. **D** The heatmap view for ZEB1 CUT&Tag signal intensity in CAFs. **E** The mean of ZEB1 CUT&Tag signals at its binding sites in indicated groups. **F** The ZEB1 CUT&Tag tracks at the locus of MYL9. **G**-**H** ChIP-qPCR (**G**) and dual-luciferase reporter assays (**H**) show that ZEB1 can bind to the MYL9 promoter region and promote MYL9 activity. **I**-**J** The results of the co-culture experiment suggested that CRC cells could promote the expression levels of ZEB1 and MYL9 genes (**I**) and proteins (**J**) in CAFs. **K** A hypothetical experimental model describing how MYL9 in CAFs promotes CRC proliferation, metastasis, and the immunosuppressive microenvironment. Co-IP, co-immunoprecipitation; ChIP, chromatin immunoprecipitation; qPCR, quantitative polymerase chain reaction; CRC, colorectal cancer; CAFs, cancer-associated fibroblasts

CCL2 secretion is regulated by the ERK 1/2 pathway [28]. In addition, TGF-β1 activate MAPK pathway during the interaction between tumor and stromal cells [29]. Therefore, we hypothesized that MYL9 could regulate the secretion of CCL2 and TGF-β1 in CAFs via the ERK 1/2 pathway. To verify this speculation, we silenced MYL9 gene in CAFs. Western blotting showed that MYL9 silencing could reduce the protein levels of CCL2 and TGF-β1, while the levels of ERK 1/2 and phosphorylated p-ERK 1/2 had no significant changes (Fig. 8A). Based on this result, we believe that MYL9 does not directly act on the ERK 1/2 pathway but may act on the pathway after binding with a certain protein. Protein spectral analysis revealed that MYL9 potentially binds to IQGAP1. IQGAP1 has five main domains (calponin-homology domain, polyproline protein–protein domain, four IQ-motif domains, Ras GAP-related domain, and Ras GAP C-terminal domain) through which it binds to other proteins and regulates the ERK 1/2 signaling pathway, which has been confirmed in multiple studies [30, 31]. To verify whether MYL9 could bind to IQGAP1, Co-IP experiments confirmed that MYL9 could bind to IQGAP1 protein (Fig. 8B). Whether MYL9 and IQGAP1 regulate the expression of CCL2 and TGF-β1 through the ERK1/2 pathway needs to be further verified. By overexpressing MYL9, silencing MYL9, and silencing IQGAP1 in CAFs, we found that silencing IQGAP1 and/or MYL9 at the same time could reduce the expression of CCL2, TGF-β1, and phosphorylated p-ERK 1/2. When MYL9 was overexpressed and IQGAP1 was silenced, the protein levels of CCL2, TGF-β1, and phosphorylated p-ERK 1/2 were not significantly changed compared with the control group (Fig. 8A, Figure S6A, B). Therefore, we believe that IQGAP1 is a key binding protein for MYL9, which regulates CCL2 and TGF-β1 secretion of CAFs by binding to IQGAP1 and acting on ERK 1/2 pathway.

### Transcription factor ZEB1 induces MYL9 expression in CAFs

At the molecular level, EMT transcription factors, including Snail, ZEB1, and Twist1, attract immunosuppressive cells through the production of chemokines or promote the expression of immunosuppressive checkpoint molecules, thus forming a tumor immunosuppressive microenvironment. Simultaneously, it can also involve other cell functions such as cell proliferation, cell apoptosis, and metastasis [32, 33]. Interestingly, our study found that MYL9 in CAFs can not only promote the EMT process of CRC, but also regulate the secretion of CCL2 and TGF-β1 through the ERK 1/2 pathway by binding to IQGAP1. Therefore, we hypothesized that there is a prior regulatory relationship between MYL9 and EMT transcription factors. First, using the TIMER 2.0 database, we evaluated the correlation between MYL9 expression and Snail, ZEB1, and Twist1. A strong correlation was found between MYL9 and ZEB1 (Figure S6C, Supplementary file 1: Table 7). Second, paraffin-embedded CRC samples were used for immunofluorescence detection to detect the co-localization of MYL9 with ZEB1. Immunofluorescence results indicated that ZEB1 and MYL9 were significantly co-located at the interface between tumor cells and CAFs, which may also indicate an interaction between MYL9 and ZEB1 in the tumor EMT or invasion process (Figure S6D). In addition, we silenced MYL9 and ZEB1 genes, respectively, and observed the co-localization relationship between MYL9 and ZEB1 through cellular immunofluorescence. The results suggested that the co-expression of ZEB1 and MYL9 was significantly weakened after silencing ZEB1 and MYL9, respectively. MYL9 expression also decreased after silencing ZEB1 (Figure S6E, F). In order to further verify the possible relationship between ZEB1 and MYL9, western blotting showed that the expression of MYL9, phosphorylated ERK1/2, CCL2, and TGF-β1 decreased with the silencing of ZEB1 (Fig. 8C).

Therefore, we investigated whether ZEB1 could regulate the function of MYL9. We used the JASPAR database to analyze the relative scores of the MYL9 promoter sequence and ZEB1. The relative scores of ZEB1 and MYL9 were > 0.8, and there may be four potential binding sites (Supplementary file 1: Table 8). At the same time, CUT&Tag technology was used to detect whether ZEB1 could combine with MYL9, and the results suggested that ZEB1 could combine with MYL9 significantly in the primary CAFs (Fig. 8D, E, F). This was verified using ChIP-qPCR and dual-luciferase reporter gene assays. According to the ChIP-qPCR results, ZEB1 could bind to the promoter regions (P3, P4) of MYL9 (Fig. 8G, Figure S6G). Dual-luciferase reporter gene assays further showed that ZEB1 could enhance the promoter activity of MYL9 (Fig. 8H). Thus, we demonstrated that the transcription factor ZEB1 could bind to MYL9 and regulate its biological function.

The malignant feedback loop between CAFs and CRC has been extensively studied. We hypothesized that CRC progression affects ZEB1 expression and MYL9 function in CAFs. Interestingly, CRC cells were co-cultured with CAFs to observe whether CRC cells cause ZEB1 and MYL9 expression in CAFs, and PCR and western blotting confirmed that co-culture with CRC cells could promote the gene levels and protein expression of ZEB1 and MYL9 in CAFs (Fig. 8I, J), and the expression of ZEB1 and MYL9 in CAFs can further promote the progression of CRC.

Therefore, MYL9 located in CAFs can interact with IQGAP1 to regulate the secretion of TGFβ1 and CCL2 through ERK 1/2, and CCL2 and TGFβ1 can not only affect the immune microenvironment of CRC, but can also promote the proliferation of CRC through the coordination of PI3K/AKT. MYL9 expression may also promote the EMT process of CRC. In addition, we found for the first time that ZEB1 can bind to MYL9 to regulate the activity of MYL9, and that ZEB1 in CAF can be produced or further promoted during CRC progression, thus forming a positive feedback effect between CAF and CRC cells (Fig. 8K).

## Discussion

In this study, we demonstrated that MYL9 expression in CRC occurs mainly in CAFs but not in tumor cells. Secondly, MYL9 in CAFs can regulate the secretion of CAFs cytokines and chemokines such as CXCL1, CCL2, IL-10, and TGF-β1, which in turn recruit M2 macrophages and inhibit the activation of dendritic cells to induce an immunosuppressive microenvironment in CRC. In addition, CCL2 and TGF-β1 can promote the progression of CRC. Mechanically, MYL9 can regulate the secretion of CCL2 and TGF-β1 of CAFs through ERK 1/2 by binding to IQGAP1, and the secreted CCL2 and TGF-β1 promote the progression of CRC through the PI3K-AKT pathway. In addition, the expression of MYL9 in CAFs can promote EMT occurrence in CRC, and the EMT transcription factor ZEB1 can bind to MYL9 to enhance its activity (Fig. 8K).

Although it has been reported that MYL9 expression in tumor tissues is closely related to tumor prognosis, the expression and prognosis of MYL9 in CRC remain controversial and require further confirmation. Using biogenic analysis, Qiu et al. [34] and Liu et al. [35] found that MYL9 and CNN1 are hub genes associated with CRC recurrence. Based on colon cancer oligonucleotide microarray data, Yan et al. [16] found that the median survival rate of colon cancer patients with low MYL9 expression was significantly reduced. However, they lacked data and basic experimental validation. In the basic study of MYL9, Feng et al. [18] found that MYL9 was highly expressed in CRC cells and combined with YAP1, thus activating Hippo signalling and promoting the proliferation, invasion, migration, and angiogenesis of CRC cells. Interestingly, using single-cell sequencing data, transcriptome data, and primary cell isolation and culture, we found that MYL9 is expressed at low levels in CRC cells and mainly plays a role in CAFs. The mechanism is that MYL9 binds to IQGAP1, regulates the secretion of CCL2 and TGF-β1 of CAFs through ERK 1/2, and these two cytokines promote the progression of CRC through PI3K-AKT pathway. According to our results, MYL9 expression in CRC tissues was significantly higher in CAFs than in tumor cells, and its upstream and downstream regulatory mechanisms in CAFs are different from those in tumor cells. It is worth emphasizing that, in clinical studies exploring molecular targets in tumors, we need to focus on the regulatory mechanisms of molecular targets in mesenchymal cells, and studies using CAFs as a model deserve more attention. In clinical studies, a single blockade of a pathway may not be therapeutically effective, possibly because of the existence of different regulatory mechanisms that target mesenchymal cells.

The TME and immunotherapy are important research directions for cancer therapy. Whether MYL9 plays a role in the TME and immunotherapy response in cancer requires further investigation. Kimura et al. [36] found that MYL9 is a ligand of CD69 and that the expression of CD69 is linked to the MYL9 network to control the depletion state of tumor-infiltrated T cells and enhance antitumor immunity. Yokoyama et al. [37] found that the MYL9-CD69 network is strongly expressed in patients with inflammatory bowel disease and plays a crucial role in the recruitment and retention of inflammatory cells. These studies also suggest that MYL9 may play a role in tumor immunity. Lv et al. [38] found that MYL9 was associated with the expression of CAFs, CD4 + T cells, macrophage infiltration, neutrophils, and dendritic cell infiltration in CRC via pan-cancer bioinformatics analysis. However, the lack of a validation cohort may have reduced the credibility of these findings. In fact, we found that MYL9 is highly correlated with CAFs because MYL9 is highly expressed in it, which is highly likely to regulate CAFs and thus affect the CRC immune microenvironment. CAFs are abundant stromal cells in the CRC TMC, and different types of CAFs play important roles in the impact of the CRC immune environment and tumor progression [39]. In this study, we found that high MYL9 expression in CAFs was strongly associated with the infiltration of high M2 macrophages, low CD4 memory resting T cells, and activated dendritic cells in the TME. The possible mechanism is the expression of CCL2, IL-10, TGF-β1, and CXCL1 in CAFs regulated by MYL9. Many studies have confirmed that these cytokines and chemokines are closely related to the recruitment of M2 macrophages, T cell activity, and inhibition of dendritic cell maturation, resulting in an immunosuppressive microenvironment in CRC. [19–22, 40]. In addition, in the study of MYL9 and tumor immunotherapy, Kim et al. [41] showed that reduced MYL9 expression in melanoma cells can improve the efficacy of NK cell-based immunotherapy. Luo et al. [42] discovered that irradiation can upregulate the expression of the tumor antigen MYL9 in A549 cells, thus improving the immunogenic regulatory effect of irradiation on non-small cell lung cancer cells and enhancing an effective strategy for anti-tumor immunity after cancer cell vaccine irradiation. Few studies have been reported on MYL9 and immunotherapy in CRC. To the best of our knowledge, this is the first study demonstrating that patients with high MYL9 expression are insensitive to immunotherapy. Thus, MYL9 is a potential target for immunotherapy.

Previous studies have shown that CAFs promote CRC development through the secretion of exosomes and cytokines. Zhou et al. [43] found that CAF-derived exosome LINC00659 promotes the progression of CRC cells through the miR-342-3p/anx2 axis. Jahangiri et al. [44] found that CAFs enhance the proliferation and metastasis of CRC SW480 cells by stimulating long non-coding RNA UCA1. TGF-β secreted by CAFs has been considered as the main inducer of EMT [29–31]. In contrast to the above studies, we found that inhibition of MYL9 expression in CAFs inhibited the EMT process in CRC. Interestingly, we found and reported for the first time that the EMT transcription factor ZEB1 can bind to MYL9 to enhance its activity. MYL9 regulates the secretion of TGF-β, thereby inducing CRC to produce EMT, and EMT transcription factor ZEB1 binds with MYL9 to promote the positive feedback loop of MYL9's effect on EMT. In our study, we demonstrated that MYL9 can regulate the secretion of some cytokines and chemokines by CAFs, which leads to CRC progression as well as the immunosuppressive microenvironment in tumors, and that MYL9 may be an important target for CRC cell communication. Communication between tumor and mesenchymal cells is a complex process, and some biomolecules can be expressed in both tumor cells and CAFs, and the cellular communication between tumor cells and CAFs and their effects on the biological phenotype of tumors deserve attention. Therefore, in clinical studies of molecular tumor targets, studies using CAFs as models are more noteworthy.

Currently, there are various animal models of CAFs that promote tumor metastasis in vivo. Ren et al. [45] injected CAFs and tumor cells into the mammary fat pads of nude mice and performed bioluminescence imaging weekly to observe the mechanism by which CAFs induce tumor growth and metastasis. Yang et al. [46] observed lung metastasis by subcutaneously injecting the two cell types into mice. Wang et al. [47] treated HCT116 cells with CM from MRC-5 cells overexpressing miR-146a-5p and miR-155-5p and injected HCT116 cells through the tail vein of nude mice to observe lung metastasis, while Fernando-Macias et al. [48] injected CAFs and HT29 cells into the pancreatic head of nude mice to observe the effect of CAFs on tumor metastasis. This study found that when human CAFs were combined with human tumor cells, liver and lung metastases were more obvious. Based on the above CAFs mouse lung metastasis model, we speculated that CAFs combined with tumor cells injected through the tail vein could observe the metastasis of CAFs in CRC. Surprisingly, we found that the lung metastatic nodules in nude mice in the ShMYL9 + LoVo group were significantly fewer than those in the LoVo and CAFs + LoVo groups. Therefore, direct co-injection of CAFs with tumor cells via the mouse tail vein may also serve as a model to observe whether CAFs promote tumor metastasis.

## Conclusion

MYL9 is predominantly expressed in the CAFs of CRC tissues and can indirectly influence tumor biology and EMT by affecting CAFs protein expression. The relationship between MYL9 expression and CRC clinical staging and immunotherapy is closer in CAFs than in tumor cells, and the potential mechanism of MYL9 action in CAFs differs from that in tumor cells. Therefore, in clinical studies exploring the molecular targets of tumors, studies using CAFs as models are of greater interest.

### Supplementary Information

**Additional file 1: Supplementary Table 1.** Information on CRC patient samples. **Supplementary Table 2.** The shRNA sequences. **Supplementary Table 3.** List of antibodies used in the experiment. **Supplementary Table 4.** The primer sequences for RT-qPCR. **Supplementary Table 5.** The primer sequences for ChIP-qPCR. **Supplementary Table 6. **Correlation analysis between MYL9 and significant gene markers of immune cells in GEPIA. **Supplementary Table 7.** Results of correlation between MYL9 and EMT transcription factors. **Supplementary Table 8.** ZEB1 and MYL9 promoter potential binding sites prediction.

**Additional file 2.** 

**Additional file 3:** **Figure S1**. MYL9 pan-carcinoma analysis. A: Prognostic analysis of MYL9 and tumor. B: The expression level and clinical staging of MYL9 in tumor and normal tissues. C: Correlation analysis between the expression level of MYL9 and the TMB and MSI of tumors. D: Analysis of differences between MYL9 expression and tumor immunoinhibitor, immunostimulator and MHC molecule. E: Analysis of MYL9 expression and tumor drug sensitivity. TMB, tumor mutation burden; MSI, microsatellite instability; MHC, major histocompatibility complex.

**Additional file 4:** **Figure S2**. Cell localization and expression level of MYL9. A: The GES132465 and GSE144735 queues suggest that MYL9 is localized in stromal cells. B: TIMER 2.0 data found that MYL9 is associated with CAFs infiltration. C: Identification of primary CAFs (Scale bar = 100μm, Red: Vimentin; Green: a-SMA). D: Immunofluorescence of primary CAFs co-localized with MYL9 (Scale bar = 100μm, Red: a-SMA; Green: MYL9). E: The level of MYL9 protein in CAFs was higher than LoVo, SW480, HCT116, and NCM460 cells. F: siRNA silencing efficiency of MYL9. CAFs, cancer-associated fibroblasts.

**Additional file 5:** **Figure S3.** High expression of MYL9 was associated with M2 macrophage infiltration. A: Correlation of MYL9 with stromalScore and immuneScore in colon cancer (COAD) and CRC (READ). B-D: Analysis of the difference and correlation between MYL9 expression and CRC immune cell infiltration in three CRC transcriptome cohorts, B: TCGA cohort, C: GEO cohort, B: Validation cohort from our own transcriptome data. E: Immunohistochemistry indicated that the positive rate of M2 macrophages in the region with high MYL9 expression was significantly higher than that of M0 and M1 macrophages (Scale Bar = 500μm and 50μm). CRC, colorectal cancer.

**Additional file 6:** **Figure S4**. CCL2 combined with TGF-β1 promoted the proliferation, migration, and invasion of CRC cells. A: qRT-PCR was used to verify the cytokine changes after MYL9 silencing. B-C: CCK-8 assay showed CCL2 and TGF-β1 promoted the proliferation of LoVo (B) and SW480 (C) cells. D: Transwell assays showed CCL2 and TGF-β1 promoted the migration and invasion of LoVo and SW480 cells (Scale Bar = 100μm). E: A mouse model of subcutaneous tumor. Each bar represents the mean ± SD of the three independent experiments. CRC, colorectal cancer; qRT-PCR, quantitative real-time polymerase chain reaction; CCK-8, cell counting kit-8; SD, standard deviation.

**Additional file 7:** **Figure S5.** Analysis of potential mechanism of action of MYL9. KEGG and GO enrichment analysis of MYL9 in TCGA cohort (A), GEO cohort (B) and Validation cohort (C).

**Additional file 8: Figure S6**. Upstream and downstream regulation mechanism of MYL9. A: siRNA silencing efficiency of IQGAP1. B: Lentivirus knockdown efficiency of MYL9 and MYL9 overexpression. C: The correlation between MYL9 and transcription factors (ZEB1, Snail, Twist1) was analyzed in the TIMER 2.0 database. D: Tissue immunofluorescence detection of MYL9 and ZEB1 co-localization analysis (Scale Bar = 200μm and 20μm). E: siRNA silencing efficiency of ZEB1. F: Silencing MYL9 and ZEB1, cellular immunofluorescence showed that the binding of MYL9 and ZEB1 was weakened in CAFs (Scale Bar = 20μm). G: Possible binding sites in the ZEB1 and MYL9 promoter regions. CAFs, cancer-associated fibroblasts.

## Data Availability

The original contributions presented in the study are included in the article/Supplementary Material. The datasets generated during and/or analysed during the current study are available from the corresponding author on reasonable request.

## Abbreviations

TME: Tumor microenvironment
CRC: Colorectal cancer
CAFs: Cancer-associated fibroblasts
OS: Overall survival
ECM: Extracellular matrix
EMT: Epithelial-mesenchymal transition
CM: Condition medium
PBS: Phosphate-buffered saline
DMEM: Dulbecco’s modified Eagle’s medium
FBS: Fetal bovine serum
IHC: Immunohistochemistry
Co-IP: Co-immunoprecipitation
DAPI: 4,6-Diamidino-2-phenylindole
HRP: Horseradish peroxidase
PBST: PBS with Tween 20
TSA: Tyramide Signal Amplification
qRT-PCR: Quantitative real-time polymerase chain reaction
CCK8: Cell counting kit-8
ChIP: Chromatin immunoprecipitation
DFS: Disease-free survival
DSS: Disease-specific survival
RFS: Relapse-free survival
PFS: Progression-free survival
HR: Hazard ratio
CI: Confidence interval
TMB: Tumor mutation burden
MSI: Microsatellite instability
TAMs: Tumor-associated macrophages
IPS: Immunophenoscore
