# Supplementary material for: MYL9 expressed in cancer-associated fibroblasts regulate the immune microenvironment of colorectal cancer and promotes tumor progression in an autocrine manner
Source: J Exp Clin Cancer Res. 2023 Nov 6;42:294. doi: 10.1186/s13046-023-02863-2 (PMC10626665; doi:10.1186/s13046-023-02863-2)
Supplement: Supplementary file 1 — Additional file 1: Supplementary Table 1. Information on CRC patient samples. Supplementary Table 2. The shRNA sequences. Supplementary Table 3. List of antibodies used in the experiment. Supplementary Table 4. The primer sequences for RT-qPCR. Supplementary Table 5. The primer sequences for ChIP-qPCR. Supplementary Table 6. Correlation analysis between MYL9 and significant gene markers of immune cells in GEPIA. Supplementary Table 7. Results of correlation between MYL9 and EMT transcription factors. Supplementary Table 8. ZEB1 and MYL9 promoter potential binding sites prediction. [file 13046_2023_2863_MOESM1_ESM.docx]

**MYL9 expressed in Cancer-associate fibroblasts regulates the immune microenvironment of colorectal cancer and promotes tumor progression through an autocrine manner**

Shenghe Deng^1,2*^, Denglong Cheng^2*^, Jun Wang^2*^, Junnan Gu^2^, Yifan Xue^2^, Zhenxing Jiang^2^, Le Qin^2^, Fuwei Mao^2^, Yinghao Cao^3#^, Kailin Cai^2#^

1. Center for Liver Transplantation, Union Hospital, Tongji Medical College, Huazhong University of Science and Technology, Wuhan, 430022, China.
2. Department of Gastrointestinal Surgery, Union Hospital, Tongji Medical College, Huazhong University of Science and Technology, Wuhan, 430022, China.
3. Department of Digestive Surgical Oncology, Cancer Center, Union Hospital, Tongji Medical College, Huazhong University of Science and Technology, Wuhan, 430022, China.

**Corresponding Author:**

Yinghao Cao, Department of Digestive Surgical Oncology, Cancer Center, Union Hospital, Tongji Medical College, Huazhong University of Science and Technology, Wuhan, 430022, China.

E-mail: d201981630@hust.edu.cn

Kailin Cai, Gastrointestinal Surgery, Union Hospital, Tongji Medical College, Huazhong University of Science and Technology, Wuhan, 430022, China.

E-mail: [caikailin@hust.edu.cn](mailto:caikailin@hust.edu.cn)

**Keywords**: MYL9, Cancer-associated fibroblasts, Colorectal cancer, immunosuppressive microenvironment, Metastasis, Epithelial-mesenchymal transition

**Funding:** This study was supported by the Interdisciplinary program of Wuhan National High Magnetic Field Center (Grant No.WHMFC202113), Huazhong University of Science and Technology, ‘the Fundamental Research Funds for the Central Universities’, HUST (2021JYCXJJ063) and the National Natural Science Foundation of China (No.82170678).

**Supplementary Data**

**Supplementary Table 1. Information on CRC patient samples**

| **Patients** | **Age** | **Gender** | **Tumor location** | | **Stage** | **Experiment** |
| --- | --- | --- | --- | --- | --- | --- |
| **1** | **66** | **Male** | **Rectal cancer** | | **T3N2M1** | **PCR/WB/IHC** |
| **2** | **54** | **Female** | **Sigmoid colon cancer** | | **T3N2M0** | **PCR/WB/IHC** |
| **3** | **87** | **Male** | **Sigmoid colon cancer** | | **T3N1M0** | **PCR/WB/IHC** |
| **4** | **71** | **Male** | **Sigmoid colon cancer** | | **T3N0M0** | **PCR** |
| **5** | **53** | **Male** | **Sigmoid colon cancer** | | **T4aN2M0** | **PCR/WB/IF** |
| **6** | **58** | **Female** | **Sigmoid colon cancer** | | **T3N0M0** | **PCR/IHC** |
| **7** | **62** | **Male** | **Hepatic Flexure of Colon** | **T3N0M0** | | **PCR** |
| **8** | **71** | **Male** | **Hepatic Flexure of Colon** | **T3N1M0** | | **PCR** |
| **9** | **79** | **Male** | **Rectal cancer** | **T3N0M0** | | **PCR** |
| **10** | **61** | **Female** | **Rectal cancer** | **T4aN2M0** | | **PCR/WB/IHC** |
| **11** | **69** | **Male** | **Rectal cancer** | **T3N1M0** | | **WB/IHC** |
| **12** | **55** | **Male** | **Sigmoid colon cancer** | **T2N0M0** | | **IF/WB/IHC** |
| **13** | **30** | **Female** | **Sigmoid colon cancer** | **T3N0M0** | | **IF/WB/IHC** |
| **14** | **71** | **Male** | **Sigmoid colon cancer** | **T3N1M0** | | **IF/WB/IHC** |
| **15** | **63** | **Female** | **Rectal cancer** | **T3N2M0** | | **IF/WB/IHC** |
| **16** | **60** | **Male** | **Sigmoid colon cancer** | **T3N2M0** | | **Isolate CAF** |
| **17** | **72** | **Male** | **Rectal cancer** | **T4N1M0** | | **Isolate CAF** |
| **18** | **66** | **Female** | **Sigmoid colon cancer** | **T3N0M0** | | **Isolate CAF** |
| **19** | **57** | **Male** | **Rectal cancer** | **T3N1M0** | | **Isolate CAF** |
| **20** | **66** | **Male** | **Rectal cancer** | **T3N2M0** | | **Isolate CAF/IF** |

CAFs, cancer-associated fibroblasts. IF, immunofluorescence. WB, western blotting. IHC, immunohistochemistry. PCR, real-time polymerase chain reaction.

**Supplementary Table 2. The shRNA sequences.**

| **si-Control** | purchased from RIBOBIO |
| --- | --- |
| **si-MYL9#1** | GTGACCGCTTCACAGATGA |
| **si-MYL9 #2** | GGCACCCATTGATAAGAAA |
| **si-IQGAP1 #3** | GCCCAGCATTACCAAGACA |
| **si-ZEB1#1** | GGCCTGAAATCCTCTCGAA |
| **si-ZEB1#2** | GAGCAAGTGTCTGAAGAAA |
| **sh-MYL9 #1** | CATTGATAAGAAAGGCAACTT |
| **sh-MYL9 #2** | CACATCCAATGTCTTCGCAAT |
| **sh-MYL9 #3** | CCGGGAGGCACCCATTGATAA |
| **CON313** | TTCTCCGAACGTGTCACGT |

**Supplementary Table 3. List of antibodies used in the experiment.**

| **Antibody** | **Company** | **Cat#** |
| --- | --- | --- |
| **MYL9** | **Abcam** | **ab191393** |
| **a-SMA** | **Abcam** | **ab7817** |
| **Vimentin** | **CST** | **5741T** |
| **PI3K** | **Abcam** | **ab151549** |
| **Phospho-PI3K** | **Affinity** | **AF3412** |
| **AKT** | **CST** | **4619T** |
| **Phospho-AKT** | **CST** | **4060S** |
| **IQGAP1** | **Proteintech** | **22167-1-AP** |
| **TGF-β1** | **Abcam** | **ab215715** |
| **CCL2** | **Abcam** | **ab214819** |
| **ERK 1/2** | **Abcam** | **ab184699** |
| **Phospho-ERK 1/2** | **Abcam** | **ab201015** |
| **E-cadherin** | **Abcam** | **ab40772** |
| **N-cadherin** | **CST** | **14215S** |
| **GAPDH** | **CST** | **5174T** |
| **ZEB1 (ChIP)** | **Proteintech** | **21544-1-AP** |
| **ZEB1** | **CST** | **3396S** |
| **CD163** | **Abcam** | **ab182422** |
| **CD83** | **Abcam** | **ab275021** |
| **CD4** | **Abcam** | **ab133616** |
| **CD45RO** | **Proteintech** | **CL488-65150** |
| **APC anti-human CD80 Antibody** | **BioLegend** | **375404 / 100 tests** |
| **PE anti-human CD163 Antibody** | **BioLegend** | **333606 / 100 tests** |

**CST: Cell Signaling Technology**

**Supplementary Table 4.** **The primer sequences for RT-qPCR.**

| **Gene** | **Forward primer (5′ - 3′)** | **Reverse primer (5′ - 3′)** |
| --- | --- | --- |
| **GAPDH** | GGCAGAGATGATGACCCTTTT | AGATCCCTCCAAAATCAAGTGG |
| **MYL9** | GGATGTGATTCGCAACGCCTTTG | GCTTGGTATGGACGGGTGTGTG |
| **IL-10** | TCATGGTGAGCACTACCTGACTAGC | TCAAAGTTCCCAAGCAGCCCTTC |
| **CCL2** | CCTTCTGTGCCTGCTGCTCATAG | TTCTTGGGTTGTGGAGTGAGTGTTC |
| **CXCL1** | CTGCTGCTCCTGCTCCTGGTAG | TTCCTCCTCCCTTCTGGTCAGTTG |
| **TGFB1** | GTTGAGTGACAGGAGGCTGCTTAG | AAAGAGGACCAGGCGGAGAAGG |
| **HGF** | TTCCATGATACCACACGAACACAGG | ACCCTCAGCCCTCAGTAAGATTCTC |
| **CCL20** | TACTCCACCTCTGCGGCGAATC | ACCCTCCATGATGTGCAAGTGAAAC |
| **CCL26** | CAGCACTTTGAGAGGCCGAGAAG | GCAGCAGGTCTTGGATATGTCACTC |
| **CCL7** | CCCTCACCCTCCAACATGAAAGC | AGCACAGATCTCCTTGTCCAGTTTG |
| **CCL8** | CTTCTGTGCCTGCTGCTCATGG | CTCCTTGCCCCGTTTGGTCTTG |
| **CX3CL1** | TCCTCTGCTGCTGGCTGGTTAG | TTGTTGGTGGTGATGGTGGTGATG |
| **IQGAP1** | CTCGCTGCCGTGGATACTTAGTTC | TCGGACCACAACCATAGGAGGATC |
| **CD11b** | ATGGATGGACTGGTAGACCTGACTG | TGTCTGTCTGCGTGTGCTGTTC |
| **CD68** | GCTACATGGCGGTGGAGTACAATG | CGATGATGAGAGGCAGCAAGATGG |
| **CD163** | GTGTGTGATGACTCTTGGGACTTGG | ACTGACGGGATGAGCGACCTG |
| **CD80** | GCCATCAACACAACAGTTTCCCAAG | GCAGTAGGTCAGGCAGCATATCAC |
| **CD86** | TCTCTGGTGCTGCTCCTCTGAAG | TGGTGGATGCGAATCATTCCTGTG |
| **ARG1** | ATTGAGAAAGGCTGGTCTGCTTGAG | ACTTGTGGTTGTCAGTGGAGTGTTG |

**Supplementary Table 5.** **The primer sequences for ChIP-qPCR.**

| **Gene** | **Forward primer (5′ - 3′)** | **Reverse primer (5′ - 3′)** |
| --- | --- | --- |
| **MYL9 CHIP PRIMER 1** | ATTCCGTTTGCCTCTCTGGA | GGGCCTTACAAGGCAGAAGA |
| **MYL9 CHIP PRIMER 2** | CATCCTCCTGACCTCCTGCT | GAGGCCTCCCACTTGTATGG |

**Supplementary Table 6** **Correlation analysis between MYL9 and significant gene markers of immune cells in GEPIA.**

| **Description** | **Gene markers** | **COAD** | | **READ** | |
| --- | --- | --- | --- | --- | --- |
|  |  | **Corrlation** | **P value** | **Correlation** | **P value** |
| **Monocyte** | CD86 | 0.31 | 2.3E-07 | 0.46 | 3.9E-06 |
|  | CD115 (CSF1R) | 0.4 | 7.8E-12 | 0.53 | 7.6E-08 |
| **TAM** | CCL2 | 0.44 | 3.6E-14 | 0.42 | 3.3E-05 |
|  | CD68 | 0.27 | 7.2E-10 | 0.33 | 0.0015 |
|  | IL10 | 0.27 | 6.3E-06 | 0.28 | 0.0077 |
| **M1 Macrophage** | INOS(NOS2) | -0.11 | 0.064 | -0.25 | 0.015 |
|  | IRF5 | 0.14 | 0.018 | 0.31 | 0.0027 |
|  | COX2(PTGS2) | 0.072 | 0.24 | 0.14 | 0.17 |
| **M2 Macrophage** | CD163 | 0.31 | 9.5E-08 | 0.47 | 1.8E-06 |
|  | VSIG4 | 0.36 | 1.1E-09 | 0.49 | 8.0E-07 |
|  | MS4A4A | 0.34 | 1.2E-08 | 0.5 | 5.2E-07 |
| **Neutrophils** | CD11b (ITGAM) | 0.34 | 7.1E-09 | 0.51 | 2.3E-07 |
|  | CCR7 | 0.18 | 0.0035 | 0.12 | 0.27 |
| **Dendritic cell** | HLA-DPB1 | 0.19 | 0.0018 | 0.5 | 3E-07 |
|  | HLA-DRA | 0.15 | 0.01 | 0.38 | 0.00018 |
|  | HLA-DPA1 | 0.14 | 0.02 | 0.38 | 0.00018 |
|  | BDCA-1(CD1C) | 0.25 | 3.4E-05 | 0.39 | 0.00014 |
|  | BDCA-4(NRP1) | 0.43 | 5.8E-14 | 0.5 | 2.8E-07 |
|  | CD11c (ITGAX) | 0.27 | 4.7E-06 | 0.31 | 0.0023 |
| **T cell (general)** | CD3D | 0.081 | 0.18 | 0.15 | 0.14 |
|  | CD3E | 0.15 | 0.011 | 0.22 | 0.033 |
|  | CD2 | 0.13 | 0.032 | 0.28 | 0.0078 |
| **CD8+ T cell** | CD8A | 0.044 | 0.47 | 0.25 | 0.019 |
|  | CD8B | 0.043 | 0.48 | 0.067 | 0.52 |

**Supplementary Table 7. Results of correlation between MYL9 and EMT transcription factors**

| **Gene** | **COAD** | **READ** |
| --- | --- | --- |
| **ZEB1** | **r=0.761** | **r=0.669** |
| **SNAI1** | **r=0.544** | **r=0.404** |
| **SNAI2** | **r=0.691** | **r=0.587** |
| **SNAI3** | **r=0.193** | **r=0.249** |
| **ZEB2** | **r=0.671** | **r=0.634** |
| **TWIST** | **r=0.717** | **r=0.694** |

| No. | Relative Score | Star | End | Strand | Predicted Sequence |
| --- | --- | --- | --- | --- | --- |
| P1 | 0.8641 | 1876 | 1886 | + | CCCGCCTGCCA |
| P2 | 0.8577 | 1579 | 1589 | + | GTCACCGGGCG |
| P3 | 0.8335 | 1382 | 1392 | + | TGCACCTCCTG |
| P4 | 0.8636 | 1250 | 1260 | + | CCCACATGGCC |

**Supplementary Table 8. ZEB1 and MYL9 promoter potential binding sites prediction**
