# Supplementary material for: MYL9 expressed in cancer-associated fibroblasts regulate the immune microenvironment of colorectal cancer and promotes tumor progression in an autocrine manner
Source: J Exp Clin Cancer Res. 2023 Nov 6;42:294. doi: 10.1186/s13046-023-02863-2 (PMC10626665; doi:10.1186/s13046-023-02863-2)

**Figure S1**: MYL9 pan-carcinoma analysis. A: Prognostic analysis of MYL9 and tumor. B: The expression level and clinical staging of MYL9 in tumor and normal tissues. C: Correlation analysis between the expression level of MYL9 and the TMB and MSI of tumors. D: Analysis of differences between MYL9 expression and tumor immunoinhibitor, immunostimulator and MHC molecule. E: Analysis of MYL9 expression and tumor drug sensitivity.

TMB, tumor mutation burden; MSI, microsatellite instability; MHC, major histocompatibility complex.


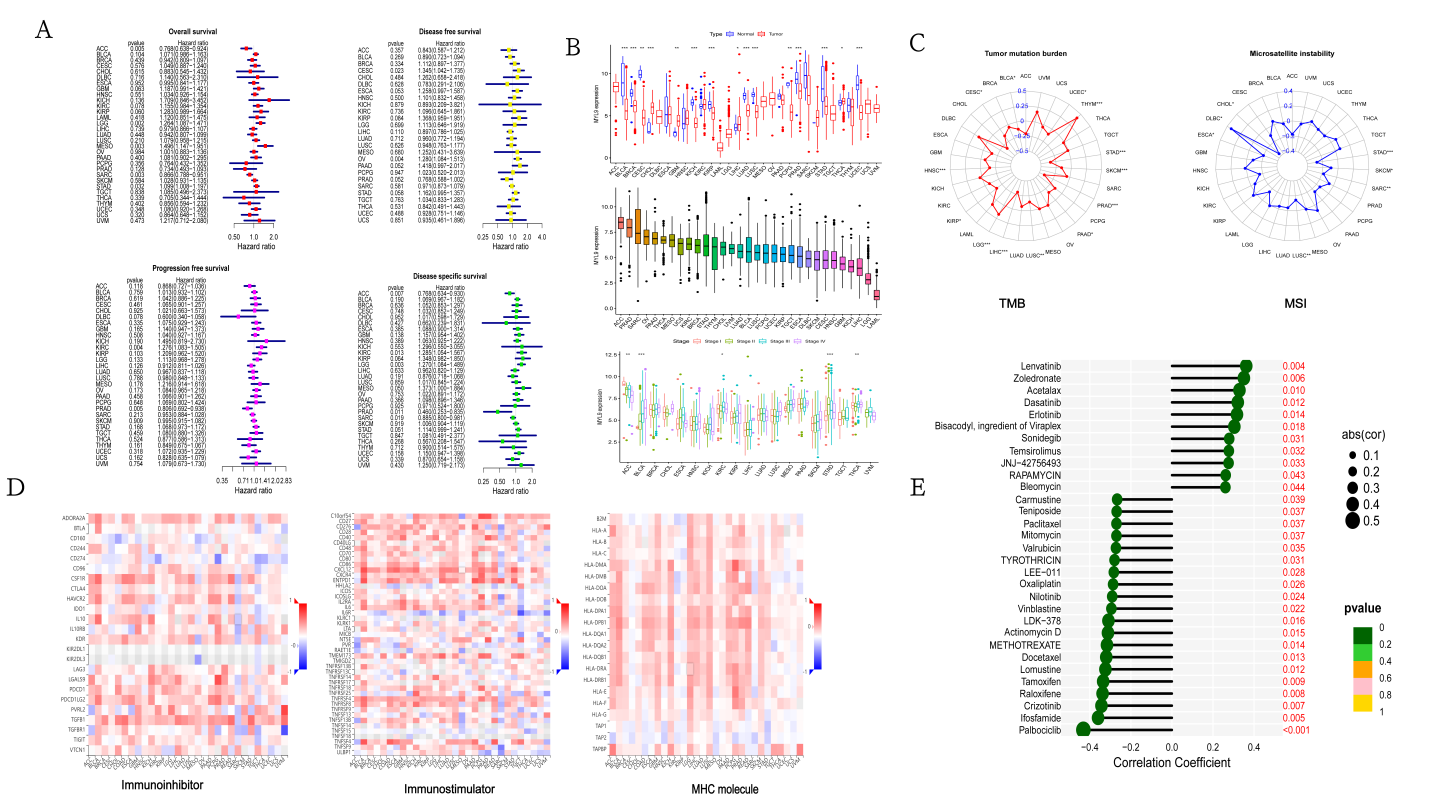

Supplement: Supplementary file 3 — Additional file 3: Figure S1. MYL9 pan-carcinoma analysis. A: Prognostic analysis of MYL9 and tumor. B: The expression level and clinical staging of MYL9 in tumor and normal tissues. C: Correlation analysis between the expression level of MYL9 and the TMB and MSI of tumors. D: Analysis of differences between MYL9 expression and tumor immunoinhibitor, immunostimulator and MHC molecule. E: Analysis of MYL9 expression and tumor drug sensitivity. TMB, tumor mutation burden; MSI, microsatellite instability; MHC, major histocompatibility complex. [file 13046_2023_2863_MOESM3_ESM.docx]
