# Supplementary material for: MYL9 expressed in cancer-associated fibroblasts regulate the immune microenvironment of colorectal cancer and promotes tumor progression in an autocrine manner
Source: J Exp Clin Cancer Res. 2023 Nov 6;42:294. doi: 10.1186/s13046-023-02863-2 (PMC10626665; doi:10.1186/s13046-023-02863-2)

**Figure S2**: Cell localization and expression level of MYL9. A: The GES132465 and GSE144735 queues suggest that MYL9 is localized in stromal cells. B: TIMER 2.0 data found that MYL9 is associated with CAFs infiltration. C: Identification of primary CAFs (Scale bar = 100μm, Red: Vimentin; Green: a-SMA). D: Immunofluorescence of primary CAFs co-localized with MYL9 (Scale bar = 100μm, Red: a-SMA; Green: MYL9). E: The level of MYL9 protein in CAFs was higher than LoVo, SW480, HCT116, and NCM460 cells. F: siRNA silencing efficiency of MYL9.

CAFs, cancer-associated fibroblasts.


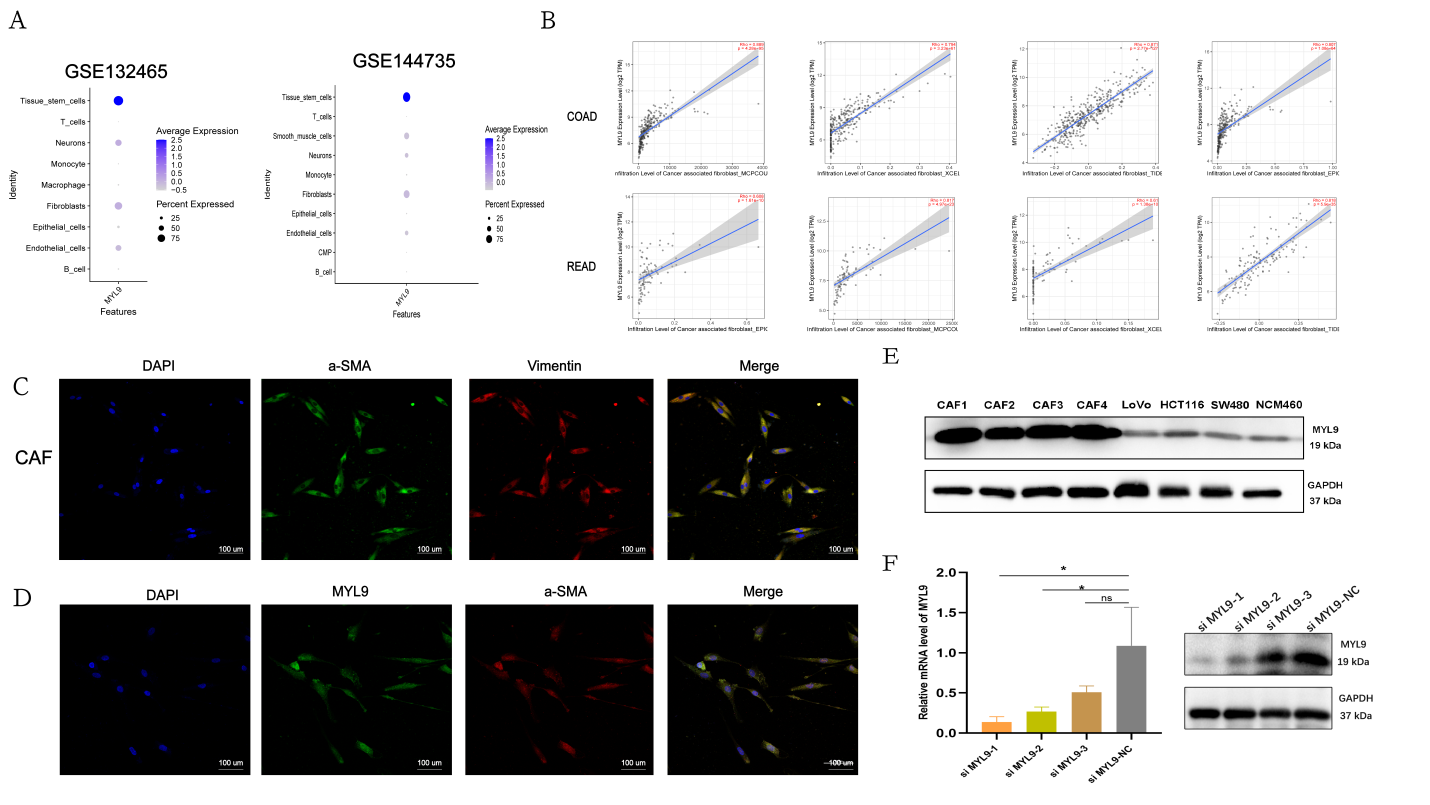

Supplement: Supplementary file 4 — Additional file 4: Figure S2. Cell localization and expression level of MYL9. A: The GES132465 and GSE144735 queues suggest that MYL9 is localized in stromal cells. B: TIMER 2.0 data found that MYL9 is associated with CAFs infiltration. C: Identification of primary CAFs (Scale bar = 100μm, Red: Vimentin; Green: a-SMA). D: Immunofluorescence of primary CAFs co-localized with MYL9 (Scale bar = 100μm, Red: a-SMA; Green: MYL9). E: The level of MYL9 protein in CAFs was higher than LoVo, SW480, HCT116, and NCM460 cells. F: siRNA silencing efficiency of MYL9. CAFs, cancer-associated fibroblasts. [file 13046_2023_2863_MOESM4_ESM.docx]
