# Supplementary material for: MYL9 expressed in cancer-associated fibroblasts regulate the immune microenvironment of colorectal cancer and promotes tumor progression in an autocrine manner
Source: J Exp Clin Cancer Res. 2023 Nov 6;42:294. doi: 10.1186/s13046-023-02863-2 (PMC10626665; doi:10.1186/s13046-023-02863-2)

**Figure S3**: High expression of MYL9 was associated with M2 macrophage infiltration. A: Correlation of MYL9 with stromalScore and immuneScore in colon cancer (COAD) and CRC (READ). B-D: Analysis of the difference and correlation between MYL9 expression and CRC immune cell infiltration in three CRC transcriptome cohorts, B: TCGA cohort, C: GEO cohort, B: Validation cohort from our own transcriptome data. E: Immunohistochemistry indicated that the positive rate of M2 macrophages in the region with high MYL9 expression was significantly higher than that of M0 and M1 macrophages (Scale Bar = 500μm and 50μm).

CRC, colorectal cancer.


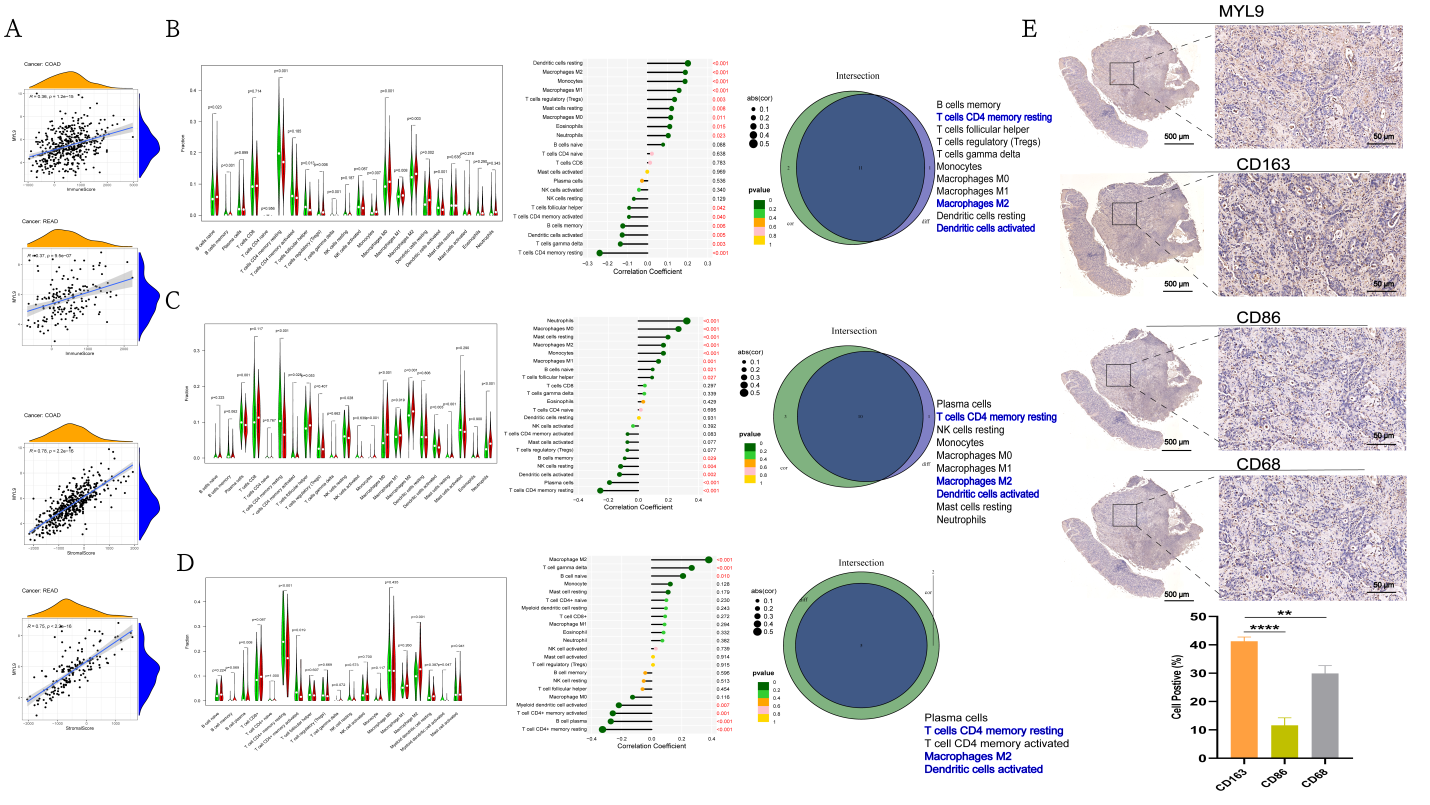

Supplement: Supplementary file 5 — Additional file 5: Figure S3. High expression of MYL9 was associated with M2 macrophage infiltration. A: Correlation of MYL9 with stromalScore and immuneScore in colon cancer (COAD) and CRC (READ). B-D: Analysis of the difference and correlation between MYL9 expression and CRC immune cell infiltration in three CRC transcriptome cohorts, B: TCGA cohort, C: GEO cohort, B: Validation cohort from our own transcriptome data. E: Immunohistochemistry indicated that the positive rate of M2 macrophages in the region with high MYL9 expression was significantly higher than that of M0 and M1 macrophages (Scale Bar = 500μm and 50μm). CRC, colorectal cancer. [file 13046_2023_2863_MOESM5_ESM.docx]
