# Supplementary material for: MYL9 expressed in cancer-associated fibroblasts regulate the immune microenvironment of colorectal cancer and promotes tumor progression in an autocrine manner
Source: J Exp Clin Cancer Res. 2023 Nov 6;42:294. doi: 10.1186/s13046-023-02863-2 (PMC10626665; doi:10.1186/s13046-023-02863-2)

**Figure S4**: CCL2 combined with TGF-β1 promoted the proliferation, migration, and invasion of CRC cells. A: qRT-PCR was used to verify the cytokine changes after MYL9 silencing. B-C: CCK-8 assay showed CCL2 and TGF-β1 promoted the proliferation of LoVo (B) and SW480 (C) cells. D: Transwell assays showed CCL2 and TGF-β1 promoted the migration and invasion of LoVo and SW480 cells. E: A mouse model of subcutaneous tumor. Each bar represents the mean ± SD of the three independent experiments.

CRC, colorectal cancer; qRT-PCR, quantitative real-time polymerase chain reaction; CCK-8, cell counting kit-8; SD, standard deviation.


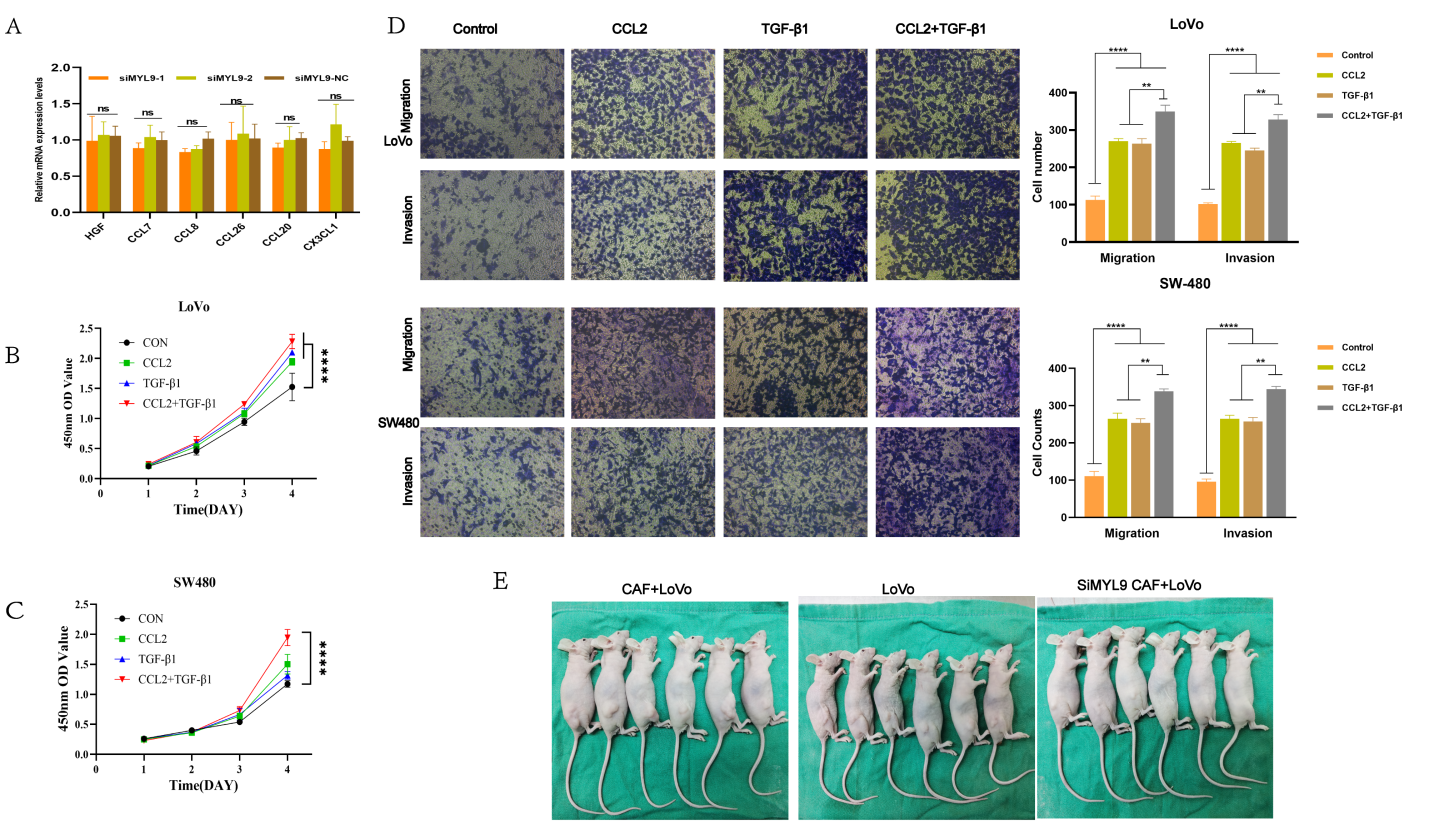

Supplement: Supplementary file 6 — Additional file 6: Figure S4. CCL2 combined with TGF-β1 promoted the proliferation, migration, and invasion of CRC cells. A: qRT-PCR was used to verify the cytokine changes after MYL9 silencing. B-C: CCK-8 assay showed CCL2 and TGF-β1 promoted the proliferation of LoVo (B) and SW480 (C) cells. D: Transwell assays showed CCL2 and TGF-β1 promoted the migration and invasion of LoVo and SW480 cells (Scale Bar = 100μm). E: A mouse model of subcutaneous tumor. Each bar represents the mean ± SD of the three independent experiments. CRC, colorectal cancer; qRT-PCR, quantitative real-time polymerase chain reaction; CCK-8, cell counting kit-8; SD, standard deviation. [file 13046_2023_2863_MOESM6_ESM.docx]
