# Supplementary material for: MYL9 expressed in cancer-associated fibroblasts regulate the immune microenvironment of colorectal cancer and promotes tumor progression in an autocrine manner
Source: J Exp Clin Cancer Res. 2023 Nov 6;42:294. doi: 10.1186/s13046-023-02863-2 (PMC10626665; doi:10.1186/s13046-023-02863-2)

**Figure S5**: Analysis of potential mechanism of action of MYL9. KEGG and GO enrichment analysis of MYL9 in TCGA cohort (A), GEO cohort (B) and Validation cohort (C).


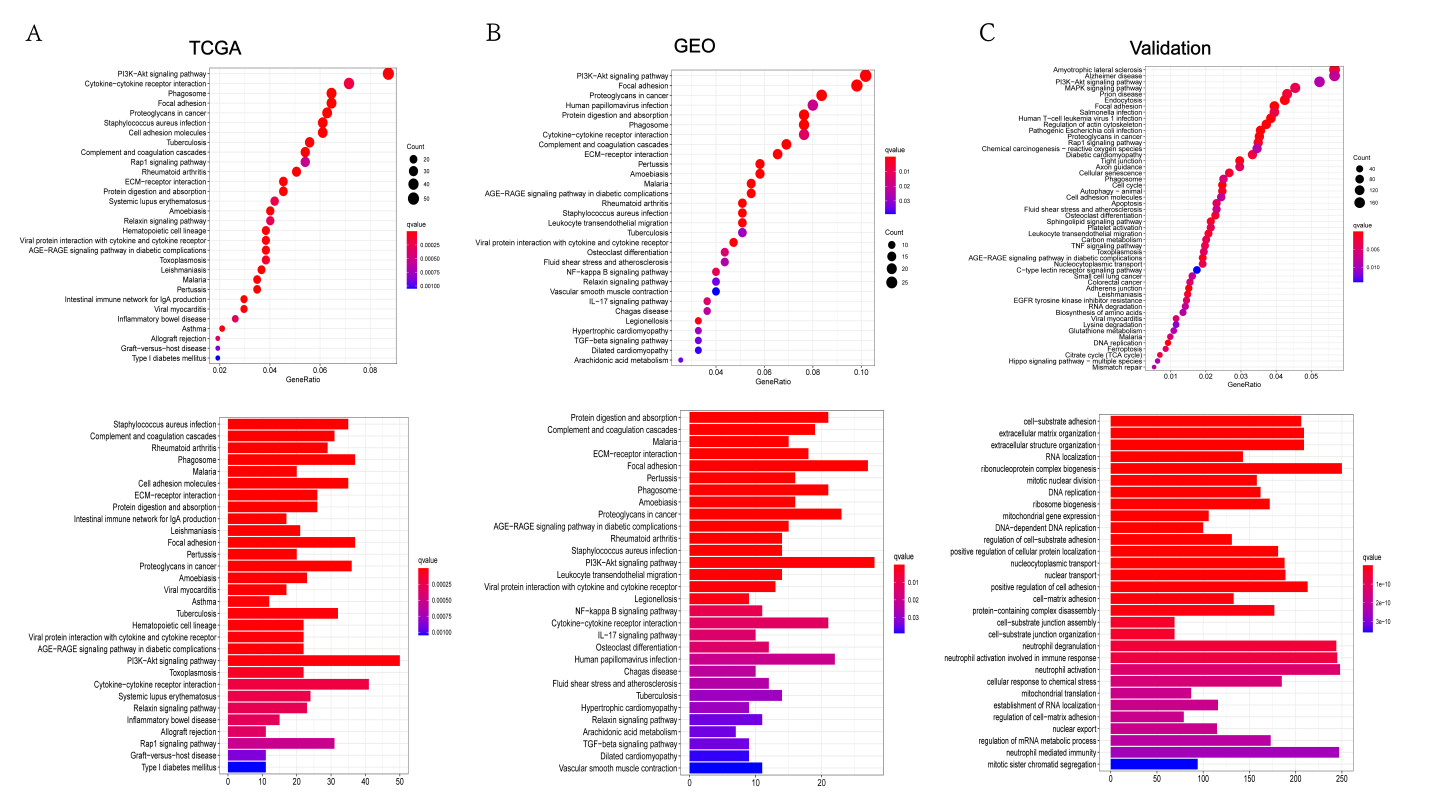

Supplement: Supplementary file 7 — Additional file 7: Figure S5. Analysis of potential mechanism of action of MYL9. KEGG and GO enrichment analysis of MYL9 in TCGA cohort (A), GEO cohort (B) and Validation cohort (C). [file 13046_2023_2863_MOESM7_ESM.docx]
