# Supplementary material for: MYL9 expressed in cancer-associated fibroblasts regulate the immune microenvironment of colorectal cancer and promotes tumor progression in an autocrine manner
Source: J Exp Clin Cancer Res. 2023 Nov 6;42:294. doi: 10.1186/s13046-023-02863-2 (PMC10626665; doi:10.1186/s13046-023-02863-2)

**Figure S6**: Upstream and downstream regulation mechanism of MYL9. A: siRNA silencing efficiency of IQGAP1. B: Lentivirus knockdown efficiency of MYL9 and MYL9 overexpression. C: The correlation between MYL9 and transcription factors (ZEB1, Snail, Twist1) was analyzed in the TIMER 2.0 database. D: Tissue immunofluorescence detection of MYL9 and ZEB1 co-localization analysis (Scale Bar = 200μm and 20μm). E: siRNA silencing efficiency of ZEB1. F: Silencing MYL9 and ZEB1, cellular immunofluorescence showed that the binding of MYL9 and ZEB1 was weakened in CAFs (Scale Bar = 20μm). G: Possible binding sites in the ZEB1 and MYL9 promoter regions.

CAFs, cancer-associated fibroblasts.


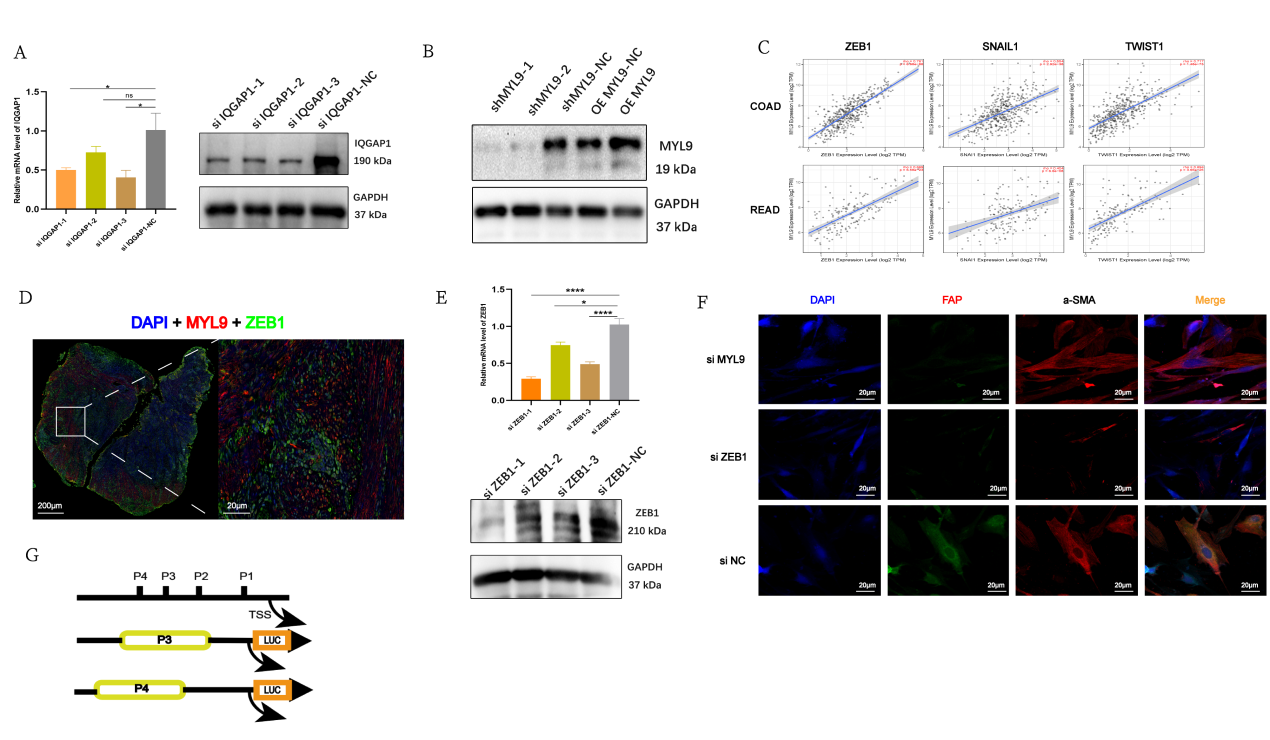

Supplement: Supplementary file 8 — Additional file 8: Figure S6. Upstream and downstream regulation mechanism of MYL9. A: siRNA silencing efficiency of IQGAP1. B: Lentivirus knockdown efficiency of MYL9 and MYL9 overexpression. C: The correlation between MYL9 and transcription factors (ZEB1, Snail, Twist1) was analyzed in the TIMER 2.0 database. D: Tissue immunofluorescence detection of MYL9 and ZEB1 co-localization analysis (Scale Bar = 200μm and 20μm). E: siRNA silencing efficiency of ZEB1. F: Silencing MYL9 and ZEB1, cellular immunofluorescence showed that the binding of MYL9 and ZEB1 was weakened in CAFs (Scale Bar = 20μm). G: Possible binding sites in the ZEB1 and MYL9 promoter regions. CAFs, cancer-associated fibroblasts. [file 13046_2023_2863_MOESM8_ESM.docx]
